# Demand creation and retention strategies for oral pre-exposure prophylaxis for HIV prevention among men who have sex with men and transgender women: a systematic review and meta-analysis

**DOI:** 10.1186/s12879-023-08693-z

**Published:** 2023-11-14

**Authors:** Nathalia Sernizon Guimarães, Laio Magno, Gabriel Marinho Bahia Monteiro, Izabel Cristina Neves Ramos, Caroline Tianeze de Castro, Thais Regis Aranha-Rossi, Marcos Pereira, Inês Dourado

**Affiliations:** 1 Faculdade Ciências Médicas de Minas Gerais, R. Basílio da Gama, Canela, Salvador, Bahia 40110-040 Brazil; 2 Fundação de apoio à Fiocruz (FIOTEC) Scholarship, Avenida, Brazil; 3 Departamento Ciências da Vida, Universidade do Estado da Bahia, Salvador, Brazil; 4 Instituto de Saúde Coletiva, Universidade Federal da Bahia, Salvador, Bahia Brazil; 5 Life Sciences Departament, Universidade do Estado da Bahia, Salvador, Bahia Brazil

**Keywords:** Demand creation to PrEP access, Retention in PrEP services, Men who have sex with men, Transgender women, Prevention of HIV infection

## Abstract

**Background:**

Men who have sex with men (MSM) and transgender women (TGW) have a disproportionately higher risk of human immunodeficiency virus (HIV) infection than other groups. Oral HIV pre-exposure prophylaxis (PrEP) is an effective prevention tool and should be offered to those at higher risk. Identifying demand creation strategies (DCS) and retention strategies (RS) to improve PrEP persistence is essential to control the HIV epidemic.

**Aim:**

We aimed to identify the (DCS and RS with higher proportions among MSM and TGW.

**Methods:**

A systematic review and meta-analysis of prospective studies were conducted, with studies retrieved from five databases until November, 2022 following the Cochrane and PRISMA guidelines. The study protocol was registered in PROSPERO (CRD42022323220). The outcomes were DCS and RS for PrEP use among MSM and TGW. Strategies used for users enrolled in the PrEP-recruited (DCS) were classified as face-to-face (peer educator recruitment at social venues, nongovernmental organizations, and parties; direct referrals by health services; friends and/or sexual partners); online (chatbot or peer educator recruitment on social media [e.g., , Instagram or Facebook] or dating/hook-up apps [e.g., Grindr, Tinder, Badoo, and Scruff]); and mixed (face-to-face and online). RS was classified as provider counseling (face-to-face by a health professional; prevention of HIV risk counseling, distribution of condoms, lubricants, and testing for HIV or other sexually transmitted infections); online counseling (text messages, chatbots, telephone calls, social media, and peer educators); and mixed (all previous strategies). Subgroup analyses were conducted for each treatment strategy. Meta-analyses were performed using the R software version 4.2.1.

**Results:**

A total of 1, 129 studies were retrieved from the five databases. After eligibility, 46 studies were included. For MSM, most DCS and RS were online at 91% (95% CI: 0.85–0.97; I^2^=53%), and 83% (95% CI: 0.80–0.85; I^2^=17%) respectively. For TGW, mixed DCS and RS were the most frequent at85% (95% CI: 0.60–1.00; I^2^=91%) and online counseling at 84% (95% CI: 0.64–0.95) compared to other strategies.

**Conclusion:**

Critical issues play. Pivotal role in increasing PrEP awareness among MSM and TGW, minimizing access gaps, and ensuring retention of PrEP services. Offering oral PrEP using online DCS and RS can reach and retain high numbers of MSM and TGW, and reduce HIV incidence in these populations.

**Supplementary Information:**

The online version contains supplementary material available at 10.1186/s12879-023-08693-z.

## Introduction

Men who have sex with men (MSM) and transgender women (TGW) are considered key populations in the HIV epidemic in many countries [1, 2]. They have a 25–34 times higher risk of HIV infection than men and women in the general population [3]. HIV pre-exposure prophylaxis (PrEP) is an effective prevention tool that should be offered to high-risk individuals [4].

PrEP effectiveness was established ten years ago, and its use is predicted to substantially decrease the number of new HIV infections [5]. The daily oral use of the pills combined with tenofovir and emtricitabine is highly effective in preventing sexual exposure to HIV infection and injection drug use (at least 74%) [6–8].

Although MSM and TGW are considered target populations for PrEP use, significant challenges exist in accessing and retaining the use of prophylaxis by these groups [9]. These include difficulties in obtaining funding for healthcare [10], lack of adequate guidance on the prevention of sexually transmitted infections (STIs) [11], low self-perception of HIV risk [12], and family issues such as lack of communication on sex and sexuality as well as lack of family support [13, 14]. Furthermore, stigma and discrimination associated with HIV and AIDS and the use of prophylactics are barriers to PrEP initiation among MSM and TGW [15, 16].

The high PrEP discontinuation rates in MSM and TGW represent a significant challenge in controlling the HIV epidemic, negatively influencing PrEP coverage [17]. A cohort study conducted in Brazil with adolescent MSM and TGW (aMSM and aTGW, respectively) aged 15–19 years indicated a 51.8% probability of discontinuation in the first year of PrEP use, with an increased risk of discontinuation in aTGW compared to aMSM [18]. Individual, structural, and logistic factors have been linked to the discontinuation of PrEP [19], which magnifies the challenge by requiring different approaches and involvement from other sectors. Similar barriers to PrEP access hinder PrEP continuation, which include low perception of risk for HIV [18–20], cost [17, 19], and difficulty navigating intricate medical systems [19]. Thus, identifying retention strategies (RS) to improve PrEP persistence among MSM and TGW is essential for controlling HIV epidemics.

Demand creation strategies (DCS) and RS for PrEP use should be developed to improve PrEP access and coverage among sexual minority adolescents. This study aimed to identify, synthesize, and determine the overall effect size through meta-analysis while critically evaluating the most effective DCS and RS for PrEP among MSM and TGW.

## Methods

This systematic review and meta-analysis followed the Cochrane Guidelines for Systematic Reviews of Interventions, and was conducted according to the Preferred Reporting Items for Systematic Reviews and Meta-Analyses (PRISMA) [21, 22]. The study protocol was registered in PROSPERO (number: CRD42022323220).

### Eligibility criteria

We included cross-sectional studies, cohort studies, and randomized control trials [RCTs] that followed MSM, regardless of sexual orientation (e.g., homosexual and bisexual), and TGW aged ≥ 18 years and assessed the PrEP DCS and/or PrEP RS.

Studies that did not describe PrEP DCS and/or PrEP RS, prior public protocols, retrospective studies, real-world settings, qualitative studies, reviews, case series, editorials (letters or commentaries), and those focused on assessing participants' intentions rather than actual PreP use, were excluded. Studies that focused on PrEP adoption intentions or interest in future PrEP use, participant’s awareness, knowledge and willingness of PrEP use, HIV risk perception, cases where participants were already using PrEP at the beginning of the research or had reported previous use of the medication (past year PrEP use), and studies specifically related to injectable PrEP use, were excluded. Furthermore, studies that assessed adherence as the only outcome, health economic evaluation (effectiveness and cost or modeling framework), ecologic studies, transgender male and female sex workers, or men who did not have sex with men or outside the theme were excluded.

### Search strategy

To answer the question “*What are the best DCS and RS for MSM and TGW on PrEP?,*” We searched five independent databases, namely PubMed/Medline, Embase, Web of Science, Central ( Cochrane Library), and Latin American and Caribbean Health Science Information (LILACS) for relevant literature. Additionally, we manually searched the reference lists of the included studies.

There were no language, date, document type, publication status, or geographic restrictions in the records. The last search was conducted in April 2022 and updated on November 2022. Descriptors were identified using the Medical Subject Headings (MeSH), *Descritores em Ciências da Saúde* (DeCS), and Embase Subject Headings (Emtree). Subsequently, they were combined with the Boolean operator “AND”, whereas their synonyms were combined with “OR”. The following meshes formed the herein-used search strategy, which was adapted based on descriptors in each database: “Pre-Exposure Prophylaxis”; “Homosexuality, Male”; “Transgender Persons”. The search strategy adopted in each database is presented in Appendix 1.

### Study selection and data extraction

Electronic search results from the defined databases were uploaded to the Rayyan Qatar Computing Research Institute [23].

The study selection and data extraction were independently performed by three investigators (NSG, GMBM, and ICNR). Any discrepancies were resolved by consensus. We adopted the following steps in the study selection: initial screening of article based on title and abstract, and thorough examination of the full-text of the selected articles. Articles that did not meet the eligibility criteria were excluded.

Information extracted from the selected studies was encoded in Excel 2019® electronic form comprising the following fields: reference, title, source, journal, impact factor, location of the study conducted, study design, follow-up period, monitoring, number of centers or health services evaluated, setting, participants’ age, population, PrEP DCS type, PrEP RS, enrolled and numbers, PrEP retention barriers, and PrEP retention facilitators.

### Quality assessment

Three investigators (NSG, GMBM, and ICNR) independently assessed the risk of bias in the selected studies according to the Joanna Briggs Institute (JBI) for determining the risk of bias. The checklists included analytical cross-sectional studies, cohort studies, and RCTs (https://jbi.global/critical-appraisal-tools). Disagreements were resolved through discussions among the three evaluators.

The overall certainty of the body of evidence was rated using the Grading of Recommendations Assessment, Development and Evaluation (GRADE) approach, while considering the overall risk of bias, consistency of effect, imprecision, indirectness, and publication bias to assess the certainty of the body of evidence [24, 25]. In the event of serious concerns in any of these domains, we rated down the quality of the evidence.

### Outcomes

The primary outcomes were the PrEP DCS and PrEP RS. Secondary outcomes were the facilitators and barriers to the retention of this population group in PrEP services.

PrEP DCS were strategies used to increase demand by delivering positive messages about the benefits of PrEP as a component of the HIV combination, and the DCS % was calculated using the following formula: people enrolled in the study/people reached by DCS. The PrEP RS was used to keep users on the PrEP services during the study period. The RS proportion was calculated as the percentage of individuals who remained in the study/the total number study / total of individuals. For the RS proportion, only studies with follow-up assessments were considered.

The PrEP DCS were classified into three groups according to Magno et al. [26]: (1) face-to-face (i.e., peer-educator recruitment at social venues, nongovernmental organizations, and parties,direct referrals by health services; friends and/or sexual partners); (2) online (i.e., chatbot or peer-educator recruitment on social media [e.g.,, Instagram or Facebook] or dating/hook-up apps [e.g., Grindr, Tinder, Badoo, and Scruff]), and mixed when both strategies were employed.

PrEP RS was used to retain users on PrEP services during the study period, which were classified as: (1) provider counseling (i.e., face-to-face by a health professional; prevention of HIV risk counseling, distribution of condoms, lubricants, and testing for HIV or other STIs; (2) online counseling (i.e., text messages, chatbot, telephone calls, social media, and peer educator); (3) cash transfer; and (4) mixed when both strategies were employed.

### Statistical analysis

We conducted a meta-analysis of the prevalence estimates that were transformed using the raw proportion (PRAW) method. The final pooled results and 95% confidence intervals (CIs) were back-transformed for ease of interpretation [27], and when the estimate for a study tended toward either 0% or 100%, the variance for that study moved toward zero. Consequently, its weight was overestimated in the present meta-analysis.

Subgroup analyses for demand creation strategies were performed considering the three strategy types (online, face-to-face, and mixed) to determine whether a strategy type could clarify our results and explain the heterogeneity. For retention, subgroup analyses were performed considering professional counseling (in-person), online counseling, and cash transfer or mixed.

A meta-regression analysis was conducted to explore the potential sources of heterogeneity for each outcome, including the study design (trial, cohort, cross-sectional), sample size (≤400, > 400), study place (Asia, Western), setting (HIV prevention and care, population), monitoring (monthly, 2–3 months, 6 months), and risk of bias (low, moderate, and high).

Forest plots were used to visually assess the pooled estimates and the corresponding 95% CIs. We calculated the Q (significance level of *p*<0.1) and I^2^ statistics, and a random-effects model was applied to assess heterogeneity.

*P*-values <0.05 were considered statistically significant in all analyses. Publication bias analysis was not performed if this measure was inappropriate for prevalence meta-analysis [28]. Analyses were performed in the R software, version 4.2.1 (R: A Language and Environment for Statistical Computing, Vienna, Austria), using the ‘Meta’ packages, versions 6.0-0.

## Results

### Search results

Our search retrieved 1,129 studies from the four selected databases. After excluding 213 duplicate articles, 916 titles and abstracts were screened. Full-text articles of the remaining 169 records were retrieved, of which 138 were excluded (Appendices 2 and 3). Additionally, through a manual search, seven studies were selected [29–35] and nine studies were updated [26, 36–43]. Therefore, 46 studies conducted between 2013 and 2022 were eligible for inclusion in this systematic review [26, 29–62, 62–70].

### Studies and users characteristics

The main characteristics of the included studies are summarized in Table 1.

**Table 1:** Characteristics of individual studies included on systematic review, 2023

**Reference**	**Country study**	**Study design**	**Follow-up**	**Population**
Grinztejn et al. 2018 [44]	Brazil	Cohort	48 weeks = 336 days	MSM (94.4%) +TGW (5.6%)
Hosek et al. 2017 [71]	United States of America	RCT	48 weeks = 336 days	yMSM (100.0%)
Songtaweesin et al. 2020 [62, 72]	Thailand	RCT	6 months = 180 days	yMSM (74.0%) + yTGW (26.0%)
Hosek et al. 2013 [45]	United States of America	RCT	24 weeks = 168 days	yMSM (100.0%)
Ferreira et al. 2018 [47]	United States of America	RCT	12 months = 365 days	ybMSM (100.0%)
Kung et al. 2018 [48]	Bangkok, Thailand	Cohort	6 months = 180 days	MSM (98.0%) + TGW (2.0%)
Lalley-Chareczko et al. 2018 [49]	United States of America	Cohort	48 weeks = 336 days	yMSM (90.0%) + yTGW (10.0%)
Liu et al. 2019 [33]	United States	RCT	36 weeks = 252 days	yMSM (96.0%) + yTGW (4.0%)
Marins et al. 2019 [50]	Brazil	Cohort	48 weeks = 336 days	MSM (94.4%) +TGW (5.6%)
Mayer et al. 2016 [51]	United States	pRCT	6 months = 180 days	MSM (100.0%)
Myers et al. 2019 [52]	United States of America	Cohort	48 weeks = 336 days	MSM (100.0%)
Rolle et al, 2017 [53]	United States of America	Cohort	24 months = 730 days	ybMSM (100.0%)
Dourado et al. 2021 [54]	Brazil	Cross-sectional	-	yMSM + yTGW
Fennel et al. 2019 [55]	United States of America	Cross-sectional	-	Young (13-24) black men who have sex with men (YBMSM)
Fields et al. 2019 [56]	United States of America	Cross-sectional	-	bMSM (100.0%)
Hoagland et al. 2017 [57]	Brazil	Cross-sectional	at 4 weeks = 28 days	MSM (94.7%) + TGW (5.3%)
Laurent et al. 2021 [58]	Abidjan (Côte d’Ivoire),Bamako (Mali), Lomé (Togo), and 119 Ouagadougou (Burkina Faso)	Cohort	-	MSM (100%) - 337 participants (57.6%) self-276 defined as bisexual
Marins et al. 2019 [50]	Brazil	Cross-sectional	at 48 weeks	MSM (94.4%) +TGW (5.6%)
Molina et al. 2017 [59]	France and Canada	Cohort	20 months = 620 days	MSM (99.0%) + TGW (1.0%)
Serota et al. 2019 [61]	United States of America	Cohort	24 months = 730 days	bMSM (100.0%)
Serota et al. 2020 [60]	United States of America	Cohort	24 months = 730 days	bMSM (100.0%)
Songtaweesin et al. 2020 [62, 72]	Thailand	Cohort	6 months = 180 days	MSM (74.0%) + TGW (26.0%)
Tun et al. 2021 [63]	Myanmar	Cohort	6 months = 180 days	MSM (92.0%) + TGW (8.0%)
Wahome et al. 2020 [64, 65]	Kenya	Cohort	12 months = 365 days	MSM (100.0%)
Wahome et al. 2020 [64, 65]	Kenya	Cohort	12 months = 365 days	MSM (100.0%)
Wheeler et al. 2019 [66]	United States of America	Cohort	52 weeks = 365 days	bMSM (100.0%)
Liu et al. 2019 [33]	United States	RCT	36 weeks = 252 days	yMSM (96%) + yTGW (4%)
Phanuphak et al. 2018 [29]	Thailand	Cohort	12 months = 365 days	MSM (86.4%) + TGW (13.6%)
Molina et al. 2015 [30]	France and Canada	RCT	9.3 months	MSM (100.0%)
Hovaguimian et al. 2022 [36]	Switzerland	Cohort	3 years	MSM (99.7%) + TGW (0.3%)
Jallil et al. 2022 [37]	Brazil	Cohort	48 weeks = 336 days	TGW (100.0%)
Magno et al. 2022 [26]	Brazil	Cross-sectional	-	MSM (92.0%) + TGW(8.0%)
Konda et al. 2022 [38]	Brazil, Mexico and Peru	Cohort	52 weeks = 365 days	TGW (100.0%)
Traikiatphum et al. 2022 [39]	Thailand	Cohort	6 months = 180 days	yMSM (100.0%)
Antoni et al. 2020 [34]	France and Canada	RCT	9 months = 283 days	MSM (?) + TGW (?)
Thongsak et al. 2022 [40]	Thailand	Cohort	6 months = 180 days	TGW (100.0%)
Wu et al. 2022 [41]	Taiwan	Cohort	4 months = 120 days	MSM (100.0%)
Lin et al. 2022 [42]	China	Cohort	3 months = 90 days	MSM (100.0%)
Mayer et al. 2020 [43]	(Austria, Denmark, France, Germany, Ireland, Italy, Netherlands, Spain, and the UK) and (Canada and the USA).	RCT	96 weeks = 672 days	MSM (98.6%) + TGW (1.4%)
Grohskopf et al. 2013 [31]	USA	RCT	24 months = 730 days	MSM (100.0%)
Kimani et al. 2021 [32]	Kenya	Cohort	6 months = 180 days	MSM (79.2%) + TGW (20.7%)
Wirtz et al. 2020 [35]	Thailand	RCT	24 months = 730 days	MSM (93.7%) + TGW (6.3%)
Dolling et al. 2016 [67]	England	RCT baseline	-	MSM (100.0%)
McCormack et al. 2016 [68]	England	RCT	24 months = 730 days	MSM (100.0%)
Schneider et al. 2021 [69]	USA	RCT baseline	55 weeks = 385 days	MSM (100.0%)
Young et al. 2018 [70]	USA	RCT	55 weeks = 385 days	MSM (100.0%)

Of the 46 included studies, 24 were cohort studies [29, 32, 36–42, 44, 48–50, 52, 53, 58–66], 16 were conducted through randomized and non-randomized clinical trials [30, 31, 33–35, 43, 45–47, 51, 62, 67–70] and six had cross-sectional design [26, 50, 54–57].

Nineteen studies were conducted in North America [31, 33, 34, 38, 45–47, 49, 51–53, 55, 56, 60, 61, 66, 69, 70]; seven in South America [26, 37, 44, 50, 54, 57], nine in Asia [29, 35, 40–42, 48, 62, 63], four in Africa [32, 58, 64, 65], and seven in Europe [30, 36, 39, 43, 59, 67, 68].

The number of health services offering PrEP included in each study ranged from 1 to 21. Six studies did not report their funding sources [40, 42, 47, 48, 62, 63].

The maximum follow-up time registered in the cohort studies and trials were as follows : 90 days [42], 120 days [41], 168 days [45], 180 days [32, 39, 40, 48, 51, 62, 63], 252 days [33], 270 days [30], 283 days [34], 336 days [37, 44, 46, 49, 50, 52], 365 days [29, 38, 47, 64–66], 385 days [69, 70], 620 days [59], 672 days [43], 730 days [31, 35, 53, 60, 61] and 1,095 days [36].

Twenty-four studies focused their assessments on MSM [30, 31, 37, 39–42, 45–47, 51–53, 55–58, 60, 61, 64–70] and nineteen focused on both population subgroups [26, 29, 32–36, 43, 44, 48–50, 50, 54, 59, 62, 63] **(**Table 1).

In total, 36,792 individuals were included in this review. Tables 2 and 3 describe the classifications of the studies based on PrEP DCS and RS outcomes. The most common DCS was face-to-face (*n*=16) (i.e., through peer educator recruitment at social venues, nongovernmental organizations, and parties; direct referrals by health services; friends and/or sexual partners), followed by online strategies (*n*=4), such as Chabot or peer-educator recruitment on social media [e.g., Instagram or Facebook] or dating/hook-up apps [e.g., Grindr, Tinder, Badoo, and Scruff]). The DCS that recruited the fewest participants was mixed (face-to-face and online) (*n*=10).

**Table 2:** Characteristics of recruitment and retention strategies in PrEP services

**Reference**	**Description of the article's strategy**	**Strategy **[26]
Grinztejn et al. 2018 [44]	In RJ, individuals seeking testing at Arco Iris, a lesbian, gay, bisexual, transgender (LGBT) non-governmental organization (NGO), and at a mobile testing unit located at a LGBT friendly venue were also assessed for potential eligibility for PrEP Brasil and subsequently referred to FIOCRUZ for screening. Social media and other media were used by the 3 sites to advertise the project and a website was constructed.	Mixed: (1) face-to-face and (2) online
Hosek et al. 2017 [71]	Multiple recruitment methods were used across sites, including street and venue-based outreach, community and school presentations, and online advertising on social media Web sites and social networking applications (apps).	Mixed: (1) face-to-face and (2) online
Songtaweesin et al. 2020 [62, 72]	voluntary HIV testing centres via counsellors, online advertising, peer recruiters and word of mouth	Mixed: (1) face-to-face and (2) online
Hosek et al. 2013 [45]	approached by study staff at community based agencies and youth venues for a brief eligibility screening via handheld personalized digital assistant	Face-to-face
Mayer et al. 2016 [51]	by community outreach by the staff of Fenway Health, a Boston community health center with expertise in sexual and gender minority health. Participants were recruited via advertisements on social media, including several sites where MSM meet sexual partners, and flyers posted within the waiting areas of Fenway Health’s clinical care sites.	Online
Myers et al. 2019 [52]	Individuals attending the clinic who were interested in participating (Most HIV-negative participants came to the clinic because they had heard about the sexual health services and about PrEP in particular. Recruitment was targeted toward sexually active YMSM of color, although the sexual health clinic was open to cisgender women and transgender women and men, and to individuals of all races and ethnicities) Those who chose to participate in the study did receive a small ($25) cash incentive to complete study assessments.	Face-to-face
Rolle et al, 2017 [53]	MSM are recruited through venue-day-time-space sampling and advertisements posted on Facebook, Grindr, and MARTA.	Mixed: (1) face-to-face and (2) online
Dourado et al. 2021 [54]	Peer-recruited by peer educators (PE) who engage with AKP at venues and schools, at online platforms/apps/transgender chatbot, a NGO/health services	Mixed: (1) face-to-face and (2) online
Fennel et al. 2019 [55]	Individuals attending the clinic who were interested in participating	Face-to-face
Fields et al. 2019 [56]	Individuals attending the clinic who were interested in participating	Face-to-face
Serota et al. 2019 [61]	Of the 298 YBMSM enrolled in EleMENt, 154 (52%) attended a PrEP clinician visit and received a PrEP prescription. At the end of follow-up, 131 (44%) reported taking a first dose of PrEP.	Face-to-face
Songtaweesin et al. 2020 [62, 72]	counselors, online advertising, peer recruiters and word of mouth.	Mixed: (1) face-to-face and (2) online
Tun et al. 2021 [63]	community-based peer educators personally and through social medias	Mixed: (1) face-to-face and (2) online
Wahome et al. 2020 [65]	10-15 peer recruiters through their personal networks and venues where sex workers meet to stablish contact with clients.	Face-to-face
Wheeler et al. 2019 [66]	peer referral, venue-based sampling, local media and word of mouth often conveyed by local health providers ir others engaged with PrEP and/or black MSM communities.	Face-to-face
Liu et al. 2019 [33]	STI screening clinic, online ads, primary care providers (participants from EPIC program)	Online
Phanuphak et al. 2018 [29]	website (www.adamslove.org) + peer recruiters	Mixed: (1) face-to-face and (2) online
Molina et al. 2015 [30]	The gay media and, especially, the Internet will be widely used with a website dedicated to the trial. A call for volunteers will be relayed by the ANRS, in the gay press and to the general public, on Internet dating sites and in clubs	Online
Jallil et al. 2022 [37]	peer referral, by our community education team, and from the HIV testing and post‐exposure prophylaxis services available at our site.	Face-to-face
Magno et al. 2022 [26]	Peer-educator recruitment through dating apps (Grindr, Badoo, Tinder and Scruff), social media (Instagram)	Online
Magno et al. 2022 [26]	face-to-face in social venues, schools, non-governmental organizations (NGO) and parties.	Face-to-face
Konda et al. 2022 [38]	social media advertisements, peer/healthcare provider referrals and through MSM/TGW peer‐educators at each site	Mixed: (1) face to face and (2) online
Wu et al. 2022 [41]	physicians; PrEP navigators	face-to-face
Lin et al. 2022 [42]	local nongovernment organizations and peer recommendations.	face-to-face
Mayer et al. 2020 [43]	Active (outreach in person or via social media) and passive (fliers, advertisements, and radio spots) recruitment methods were customised for local cultural context and language by site.	Mixed: (1) face to face and (2) online
Grohskopf et al. 2013 [31]	recruitment on 3 sites	face-to-face
Kimani et al. 2021 [32]	Through a partnership with the community-based organization AMKENI supporting MSM and TGW members, participants were contacted to enroll in the PrEP cohort	face-to-face
Wirtz et al. 2020 [35]	Community engagement principles and mobilization activities are intended to create an enabling HIV prevention environment for young MSM and TGW. For this study, our partners SWING and RSAT serve as the initial conduit for community engagement. These partners have extensive experience in providing HIV prevention programs to gay, bisexual, transgender, and other MSM, including those who exchange sex. The chief community mobilization activity is an ongoing series of forums targeting various venue types, age groups, and geographic subzones that are led by CBO staff in conjunction with young MSM and TGW. Point persons at participating venues who introduce the study also aid in raising awareness about and invite participants to community engagement activities.	face-to-face
McCormack et al. 2016 [68]	.Potentially eligible GMSM were identified during routine attendances at 13 sexual health clinics in England, 8 in London and 5 outside. Participants with regular sexual partners (in the opinion of the potential volunteer) who also met eligibility requirements were encouraged TO ENROL. Posters and electronic screens in participating sexual health clinics, as well as advertisements on social media, helped to promote the study. Business cards and leaflets advertising the study were also handed out by community organisations during outreach activities, including efforts to raise awareness of PrEP amongst GMSM. There was no financial payment for participants joining the study, nor were travel costs or other expenses paid for.	Mixed: (1) face to face and (2) online
Schneider et al. 2021 [69]	respondent-driven sampling	face-to-face
Young et al. 2018 [70]	respondent-driven sampling	face-to-face

**Table 3:** Characteristics of retention strategies at PrEP services

**Reference**	**Description of the article's strategy**	**Class**
Grinztejn et al. 2018 [44]	interactive text messages + brief paper questionnaire on mobile phone use and texting practices	Online counseling
Marins et al. 2019 [50]	interactive text messages + brief paper questionnaire on mobile phone use and texting practices	Online counseling
Hosek et al. 2017 [71]	All participants received a comprehensive package of HIV prevention services at each visit (i.e., risk reduction counseling, condoms, symptomatic STI screening and treatment) and met with a study counselor to complete an Integrated Next Step Counseling (iNSC) session.21 The iNSC approach includes exploration, problem solving and skills building around non-biomedical strategies to prevent HIV, and for those receiving PrEP, assesses participant’s desire to remain on PrEP and strategies to improve or maintain adherence. At each study visit, participants completed behavioral assessments via audio computer-assisted self-interview (ACASI), received condoms, and were dispensed study drug. Participants were provided compensation for each study visit as determined by each local IRB. $50 per visit for transportation	Professional counseling + cash transfer
Songtaweesin et al. 2020 [62, 72]	*n*=100 to receive youth friendly services (YFS) + *n*=100 YFS plus use of a PrEP adherence supporting mobile phone app (YFS + APP). Voucher: 100 points = $3	Mixed = professional counseling + online counseling + cash transfer
Hosek et al. 2013 [45]	All participants received a comprehensive package of HIV prevention services at each visit (risk reduction, condoms, sexually transmitted infection (STI) screening, treatment, etc). Behavioral assessments were conducted via audio computer-assisted self-interview at each study visit along with risk reduction counseling, condom distribution, and drug dispensation. No specific adherence counseling was offered to participants because insuring the ability to analyze for product efficacy was not an aim of the study. Participants were provided a $50 incentive for each study visit and fare for round-trip public transportation.	Mixed = Professional counseling + cash transfer
Ferreira et al. 2018 [47]	monthly check-in booster calls	Online counseling
Kung et al. 2018 [48]	N.I.	N.I.
Lalley-Chareczko et al. 2018 [49]	All participants received standard HIV-prevention services including condom provision, risk reduction counseling, HIV testing, and STI screening and treatment in addition to basic medical care. In accordance with CDC guidelines, participants were screened for HIV, STIs, and renal function at baseline and every 3 months during the study protocol period. Participants were also offered rapid tests for HIV on a monthly basis. Treatment was provided for STIs diagnosed during this study, and nonimmune subjects were offered vaccines against hepatitis A and B. Study staff provided standard of care adherence counseling to all participants at each visit.	Professional counseling
Liu et al. 2019 [33]	SMS-based adherence support + HIV testing was completed at each visit per clinic protocol. Urine specimens and rectal and pharyngeal swabs were tested quarterly for Neisseria gonorrhoeae and Chlamydia trachomatis using a nucleic acid amplification test. Participants were provided $30 at each visit for completion of study procedures.	Mixed = Professional counseling + online + cash transfer
Marins et al. 2019 [50]	interactive text messages + brief paper questionnaire on mobile phone use and texting practices	Online counseling
Mayer et al. 2016 [51]	counseling session	Professional counseling
Myers et al. 2019 [52]	A clinician performed adherence counseling as part of each clinic visit. PrEP counseling was modeled on existing clinic practices for counseling HIV-infected patients on antiretroviral adherence. Brief counseling at each visit encompassed 4 steps—assessment of adherence and factors related to it, strengthening of the therapeutic alliance, and recommendation of targeted interventions as appropriate (eg, alarm setting, pill boxes, treatment for substance use, or mental health disorders).	Professional counseling
Rolle et al, 2017 [53]	All participants receive comprehensive HIV/STI risk-reduction counseling which includes the provision of condoms and lubricants. Free transportation to all visits is available for all participants. And are invited to sign-up for medication reminders using free electronic apps and reminder services. Participants with health insurance use their insurance plan to pay for TDF/FTC and manufacturer co-pay cards are provided by the study to minimize associated prescription costs. Study staff provide assistance for uninsured men to enroll in the manufacturer patient assistance program (PAP) and receive TDF/FTC free of charge. One month after PrEP initiation, study clinicians phone participants to ensure that they have obtained TDF/FTC and perform an initial adherence and safety assessment.	Professional counseling
Laurent et al. 2021 [58]	At each scheduled visit, participants received a medical interview and examination, HIV testing, screening and treatment for other STI (syndromic approach), peer-led counselling (prevention and adherence) and psychosocial support, condoms and lubricants, and a refill of their PrEP prescription. Blood creatinine was measured at enrolment and every six months thereafter. Vaccination against hepatitis B was offered to participants who tested negative for both HBsAg and anti-HBs. Finally, peer-educators contacted participants by phone (with prior consent) if they were 15 days late for their scheduled visits. All services were free of charge. Participants were compensated US$5 for transport costs for each scheduled follow-up visit.	Mixed = Professional counseling + peer-educators + cash transfer
Molina et al. 2017 [59]	At every visit, we offered participants a comprehensive package of prevention services as previously described,13 including face-to-face risk-reduction counselling done by a peer community member, free condoms and gel, and testing and treatment of STIs. Postexposure prophylaxis was also available at study sites free of charge in case of unprotected exposure to a partner possibly infected with HIV, but only if PrEP had not been taken as recommended, because of the favourable results of the placebo-controlled phase of the study. We hired peer counsellors from the AIDES advocacy group. Each counsellor was responsible for 50 participants and was also tasked with recruitment in gay venues (eg, bars, saunas, and sex clubs). Each counsellor gave their professional mobile phone number and email to participants and could be contacted anytime to answer participants’ questions. Peer counsellors reminded participants of their appointments at study sites and to complete questionnaires just before the visits (or helped them on site when this was not done before). When the medical team needed participants to come back for the treatment of an STI or for an abnormal blood test (although the reason why participants had to come back was not known to the peer counsellors) peer counsellors contacted participants. Peer counsellors also attended all participants’ visits on site and provided adherence and risk-reduction counselling.	Professional counseling
Songtaweesin et al. 2020 [62, 72]	youth friendly services (YFS) or YFS plus use of a PrEP adherence supporting mobile phone app (YFS + APP) + telephone contact at months 2, 4 and 5 + counselling online and face-to-face + condoms and lubricants offered + STI screening.	Mixed = Professional counseling + peer-educators + cash transfer
Tun et al. 2021 [63]	counselling from peer-educators	Peer-educators
Wahome et al. 2020 [65]	during the visits, participants were counselled about benefits and risks of PrEP and the importance of adherence. During monthly visits, participants taking PrEP completed a questionnaire on PrEP knowledge, motivation to PrEP and PrEP adherence via audio computer-assisted self-interview (ACASI). Retention was acessed by refills and a one-time dried blood spot sample was collected for assessment of substance concentration levels.	Mixed = Professional counseling + online counseling
Wahome et al. 2020 [64]	during the visits, participants were counselled about benefits and risks of PrEP and the importance of adherence. During monthly visits, participants taking PrEP completed a questionnaire on PrEP knowledge, motivation to PrEP and PrEP adherence via audio computer-assisted self-interview (ACASI). Retention was acessed by refills and a one-time dried blood spot sample was collected for assessment of substance concentration levels.	Mixed = Professional counseling + online counseling
Wheeler et al. 2019 [66]	counselling, care coordination, referrals and follow-up care (C4 Intervention - sessions at each visit) + AEs (Assessment for adverse events) at each follow-up visit + measure of. 52 weeks of follow-up.	Professional counseling
Liu et al. 2019 [33]	SMS-based PrEP support intervention (Prepmate) + risk assessment, PrEP education and brief adherence counselling by health educator. Adherence measured by blood spots and STI screening.	Mixed = Professional counseling + online
Phanuphak et al. 2018 [29]	counselling, visit reminders via social media, and calls every week for a month and then 1 month after that if participants didn't show up at a follow-up visit	Mixed = Professional counseling + online
Molina et al. 2015 [30]	pill count + adherence counselling, HIV testing and biochemichal analyses. Before each visit, participants were asked to complete at home a computer-assisted structured interview to collect information about sociodemographic characteristics, use of alcohol and recreational drugs, sexual behavior, and adherence to preexposure prophylaxis during their most recent sexual intercourse.	Professional counseling
Hovaguimian et al. 2022 [36]	counselling + STI screening	Professional counseling
Jallil et al. 2022 [37]	risk reduction counselling and clinical and safety laboratory evaluations (including HIV testing and pooled or individual HIV viral load) at every study visit. As needed, TGW had access to mental health and endocrinological care and could receive FHT available at the site (estradiol valerate 2 mg plus spironolactone 100 mg), with doses adjusted by the study endocrinologist.	Professional counseling
Konda et al. 2022 [38]	Refills of PrEP and assessment concerning sexual behavior at enrollment and quarterly visits	Professional counseling
Traikiatphum et al. 2022 [39]	All participants were advised to download the mobile application “Raincoat”, produced by Focal Intelligence Co., Ltd. Based on the information-motivation-behavioral (IMB) skills model, which consists of (1) information need related to HIV prevention (2) motivation of attitudes and intentions for HIV prevention by self-assessment of HIV risk acquisition, and (3) behavioral skills necessary for HIV prevention. It is aimed to support participants to regularly assess their HIV acquisition risk and to provide a schedule of taking ED-PrEP and appointment reminders. The application included a self-evaluation feature for users to log numbers of sex acts, sexual partners, pills taken, and condom use, which were used to auto calculate HIV risk. To facilitate ED-PrEP use, participants could record when they take the first two pills, which would then auto-generate reminders for the next two pills as notifications on their mobile phone. Reminder messages were customizable, not explicitly mentioning PrEP or HIV or taking medicine but a sentence such as “We have an appointment,” and users were able to set a password to ensure privacy. The Raincoat application was available on both Android OS and iOS mobile platforms.	Online counseling
Antoni et al. 2020 [34]	HIV testing on clinical visit	Professional counseling
Thongsak et al. 2022 [40]	HIV testing and laboratorial exams on clinical visit	Professional counseling
Wu et al. 2022 [41]	social media app (UPrEPU app’s) + visit included rapid testing for HIV antigen and antibodies	Mixed = Professional counseling + online
Mayer et al. 2020 [43]	HIV testing; Sites provided local standard-of-care risk reduction counseling, adherence counseling, and condoms and lubricant. Treatment for STIs and HIV post-exposure prophylaxis was offered as per local guidelines.	Professional counseling
Grohskopf et al. 2013 [31]	Visits included AE assessment, symptom-directed physical examination, blood and urine collection, sexually transmitted infection testing, behavioral assessment via audio computer–assisted self-interview, and risk reduction and adherence counseling.	Mixed = Professional counseling + online
Kimani et al. 2021 [32]	Participants received 350 KSh (3.5 US$) to cover participation costs for each scheduled visit. PrEP initiation and maintenance followed national guidelines, including a one-month PrEP-supply (irrespective of creatinine result); a two-month supply for the next two months, and a quarterly supply thereafter. Participants were reminded of their upcoming clinic visit 24 hours before the visit date. Physical tracing was done for those who did not attend their assigned visit or who were unreachable on phone. At each visit, participants were provided with standardized PrEP adherence counselling, supporting participants to take PrEP at a regular moment in the day and discussing possible adherence challenges. Participants who had stopped taking PrEP were encouraged to re-start.	Professional counseling + cash transfer
Wirtz et al. 2020 [35]	Participants are offered a modest reimbursement for travel and their time, 800 Thai Baht (THB) per session (approximately US $25)	Cash transfer
McCormack et al. 2016 [68]	attend clinic every 3 months + HIV and STI testing	Professional counseling
Schneider et al. 2021 [69]	digital network (by Facebook) - (1) HIV facts and myths; (2) background on PrEP; (3) role playing conversations about motivating peers to engage in PrEP care; and (4) leveraging social media to spread awareness about PrEP.	Online
Hoagland et al. 2017 [57]	interactive text messages + brief paper questionnaire on mobile phone use and texting practices	Online

The retention of health services providing PrEP was observed in 28 studies. The retention strategies were online counseling (text messages, chatbot, telephone calls, social media, and peer-educator) [39, 44, 69], mixed strategy [29, 31–33, 41, 45, 46, 58, 62, 64], provider counseling (face-to-face by a health professional; prevention to HIV-risk counseling, distribution of condoms, lubricants, and testing for HIV or other STIs) [30, 36–38, 40, 43, 49, 51–53, 66, 68], peer educators [63],and cash transfer [35]. The last two alone.

### Quality assessment

Of the 46 studies included in this systematic review, 29 were evaluated and included in the meta-analysis. Regarding DCS outcomes, six observational studies and four trials presented a high risk of bias, seven observational studies and six trials were identified as having a moderate risk of bias, and four observational studies and two trials were identified as having a low risk of bias. Regarding retention outcome, one observational study and three trials presented a high risk of bias, 11 observational studies and six trials were identified as having a moderate risk of bias, and four observational studies and two trials were identified as having a low risk of bias. The individual studies’ risks of bias for each study are presented in Appendices 4–7.

### Meta-analysis results

#### Prevalence of users enrolled in PrEP recruited by DCS

**Fig. 1:**
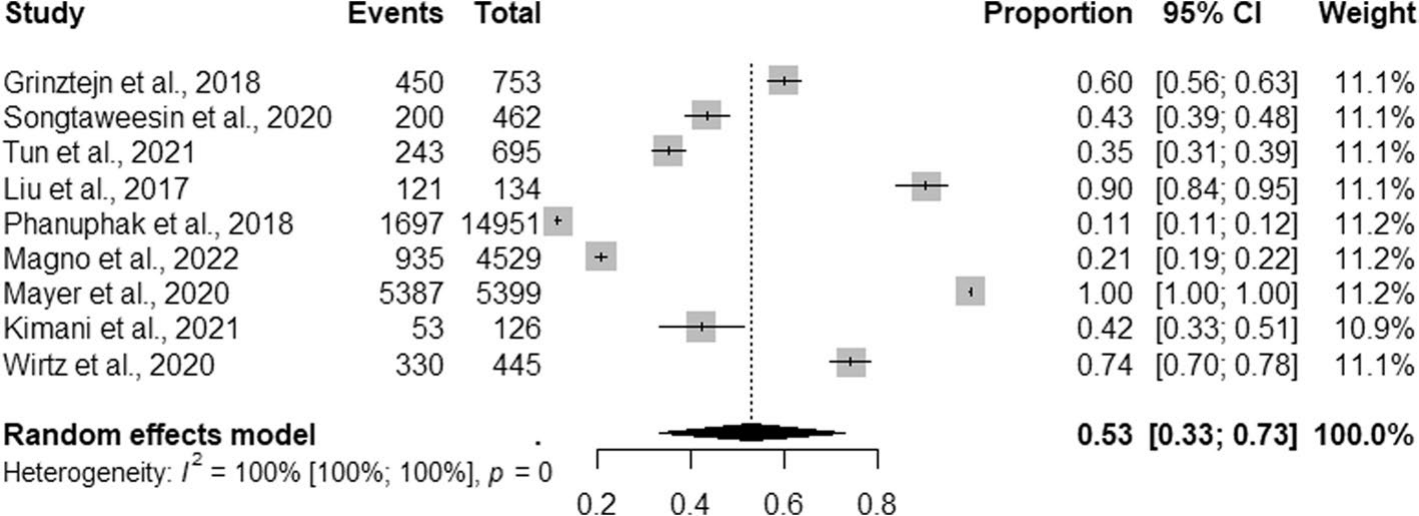
Forest plot of pooled proportions of MSM and TGW enrolled in PrEP DCS (*n*=09)

The overall prevalence of users enrolled in PrEP recruited by demand creation strategy among the entire sample evaluated was 53% (95% CI: 0.33–0.73) with a high level of heterogeneity (I ^2^=100%) (Fig. 1).

**Fig. 2:**
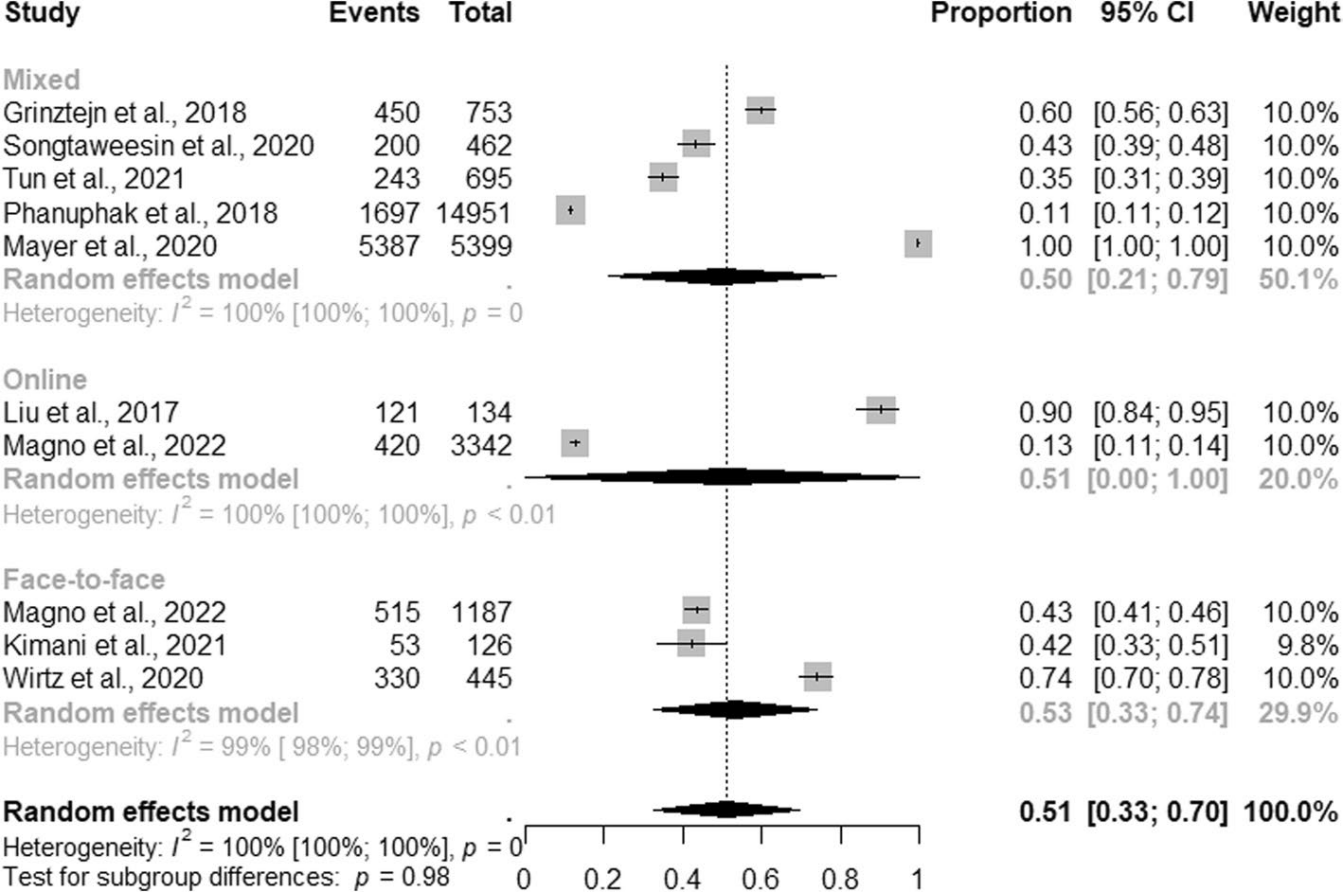
Forest plot of pooled proportions of PrEP DCS among the entire sample (MSM and TGW) (*n*=10 report and 09 studies because of difference strategies)

The analysis of subgroups by types of PrEP DCS for the overall population revealed that face-to-face, online, and mixed recruited 53% (95% CI: 0.33–0.74; I^2^=100%); 51% (95% CI: 0.00–1.00; I^2^=100%); 50% (95% CI: 0.21–0.79; I^2^=100%), of the population respectively (Fig. 2).

**Fig. 3:**
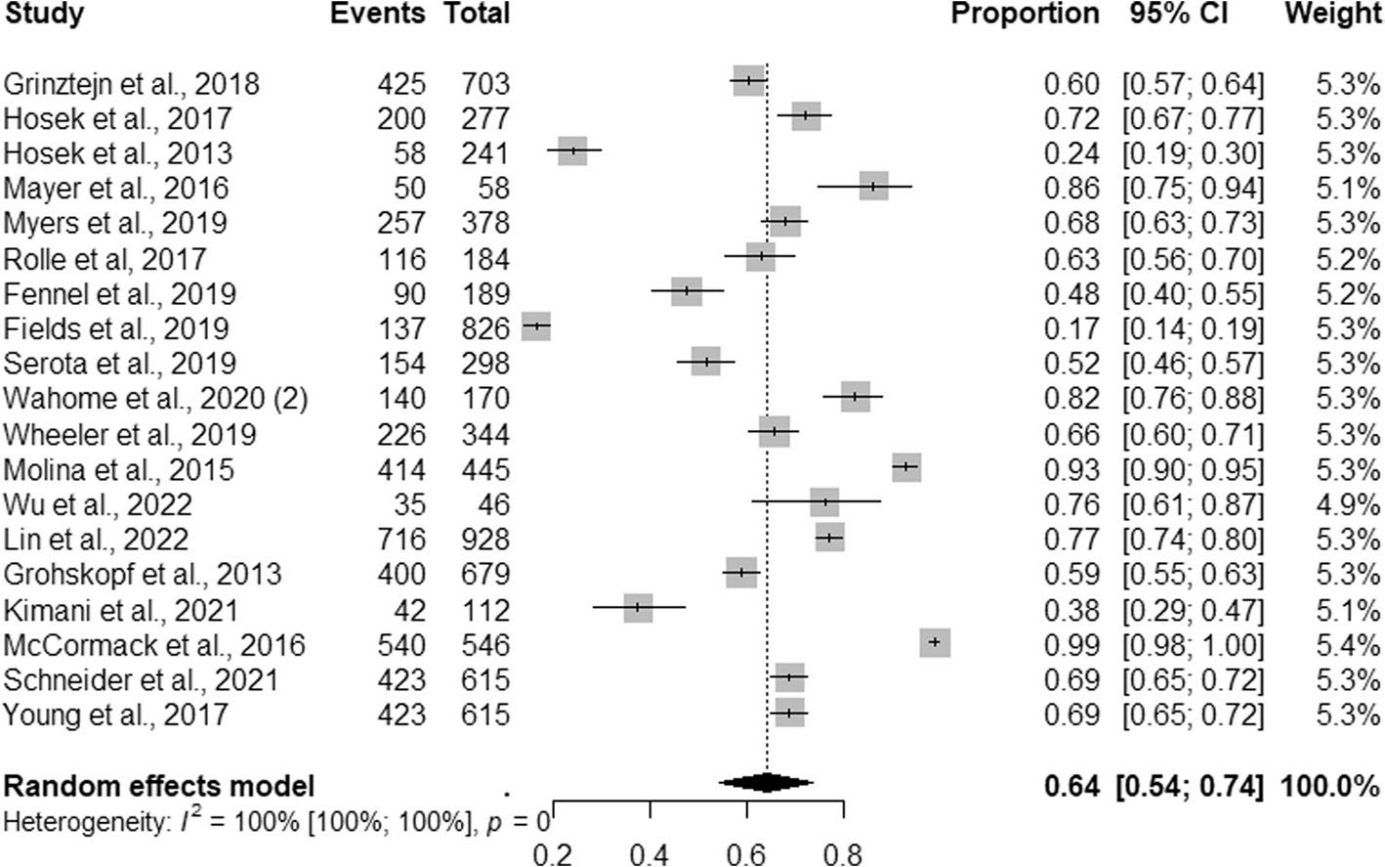
Forest plot of pooled proportions of PrEP DCS among MSM (*n*=19)

Of the 36 included studies, 19 assessed the percentage of MSM users enrolled in PrEP recruited. The combined proportion of MSM was 64% (95% CI: 0.54–0.74; I^2^=100%) (Fig. 3).

**Fig. 4:**
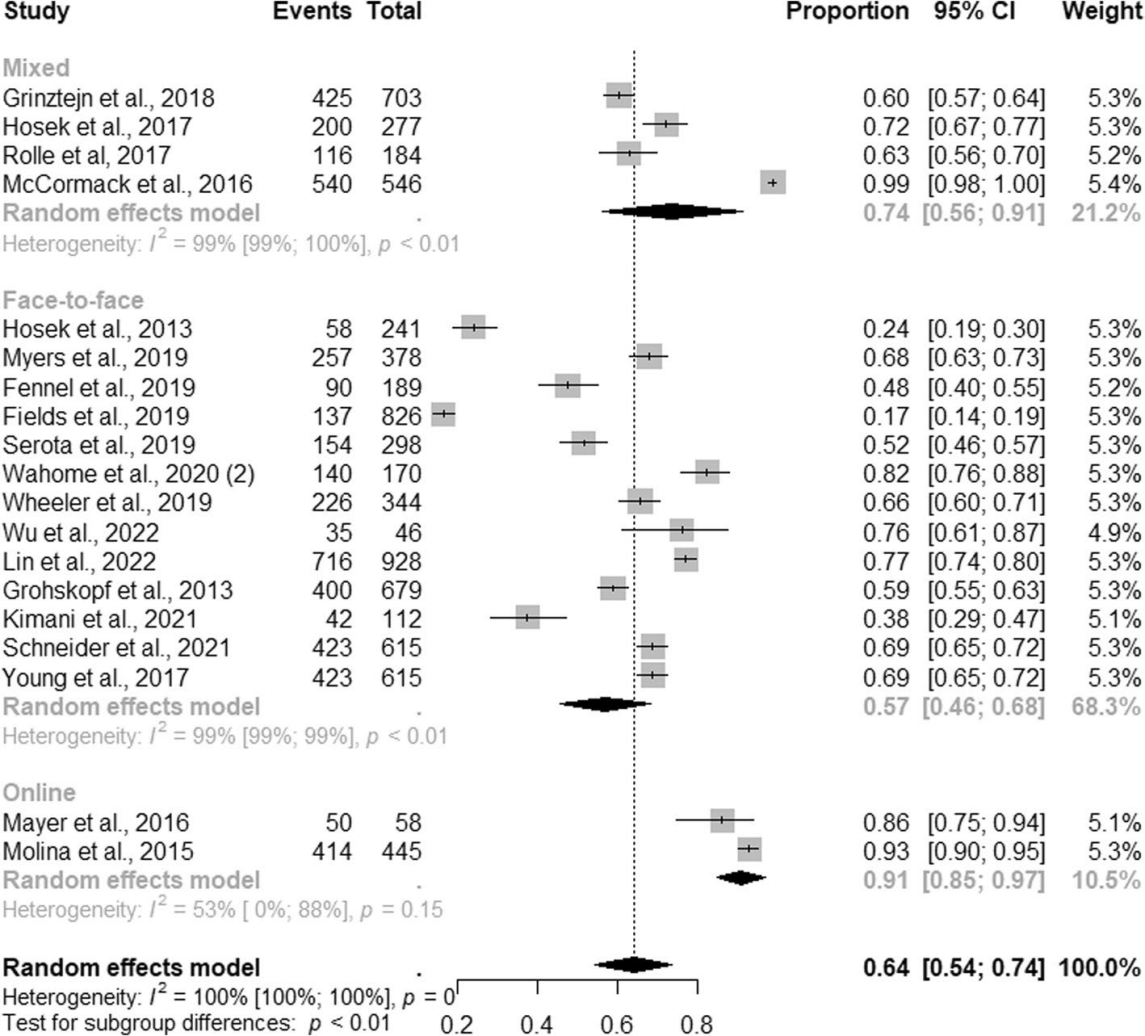
Forest plot of pooled proportions of PrEP DCS among MSM (*n*=19)

Among the studies which discriminated against the number of MSM users enrolled in PrEP recruited by demand creation strategy, four, thirteen, and two studies evaluated mixed, face-to-face, and online DCS, respectively. The subgroup analysis by DCS type showed that 91% of MSM (95% CI: 0.85–0.97; I^2^=53%) were recruited through online, 74% (95% CI: 0.56–0.91; I^2^=99%) through mixed, and 57% through face-to-face (95% CI: 0.46–0.68; I^2^=99%) strategies **(**Fig. 4).

**Fig. 5:**
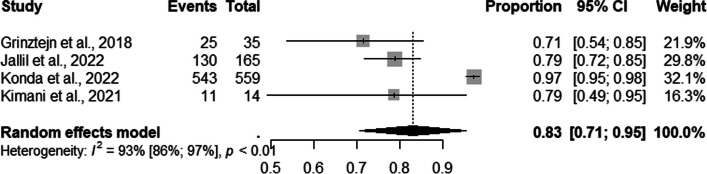
Forest plot of pooled proportions of PrEP DCS among TGW (*n*=04)

Regarding TGW, four studies presented information on users enrolled in PrEP recruited. The pooled proportions of DCS for PrEP use among TGW was 83% (95% CI: 0.71–0.95; I ^2^=100%) (Fig. 5).

**Fig. 6:**
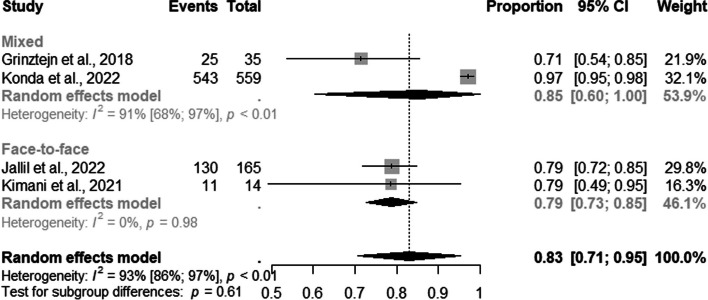
Forest plot of pooled proportions of PrEP DCS among TGW (*n*=04)

In the subgroup analysis by DCS, we observed that 85% of the TGW were recruited via mixed(95% CI: 0.60–1.00; I^2^=91%) and 79% via face-to-face (95% CI: 0.73–0.85) strategies (Fig. 6).

### PrEP retention strategies

**Fig. 7:**
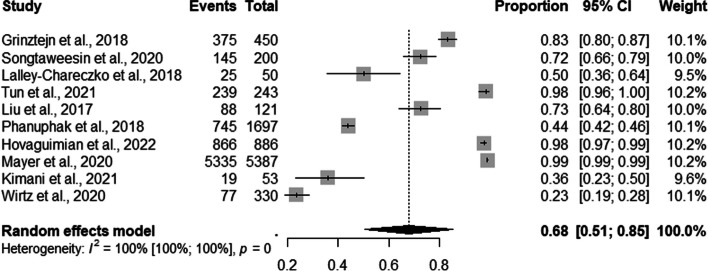
Forest plot of pooled proportions of retention to pre-exposure prophylaxis (PrEP) service among the entire sample (MSM and TGW) (*n*=10)

Ten studies assessed the prevalence of PrEP service retention in the overall sample. The global estimate of prevalence was 68% (95% CI: 0.51–0.85) with a high level of heterogeneity (I^2^=100%) (Fig. 7).

**Fig. 8:**
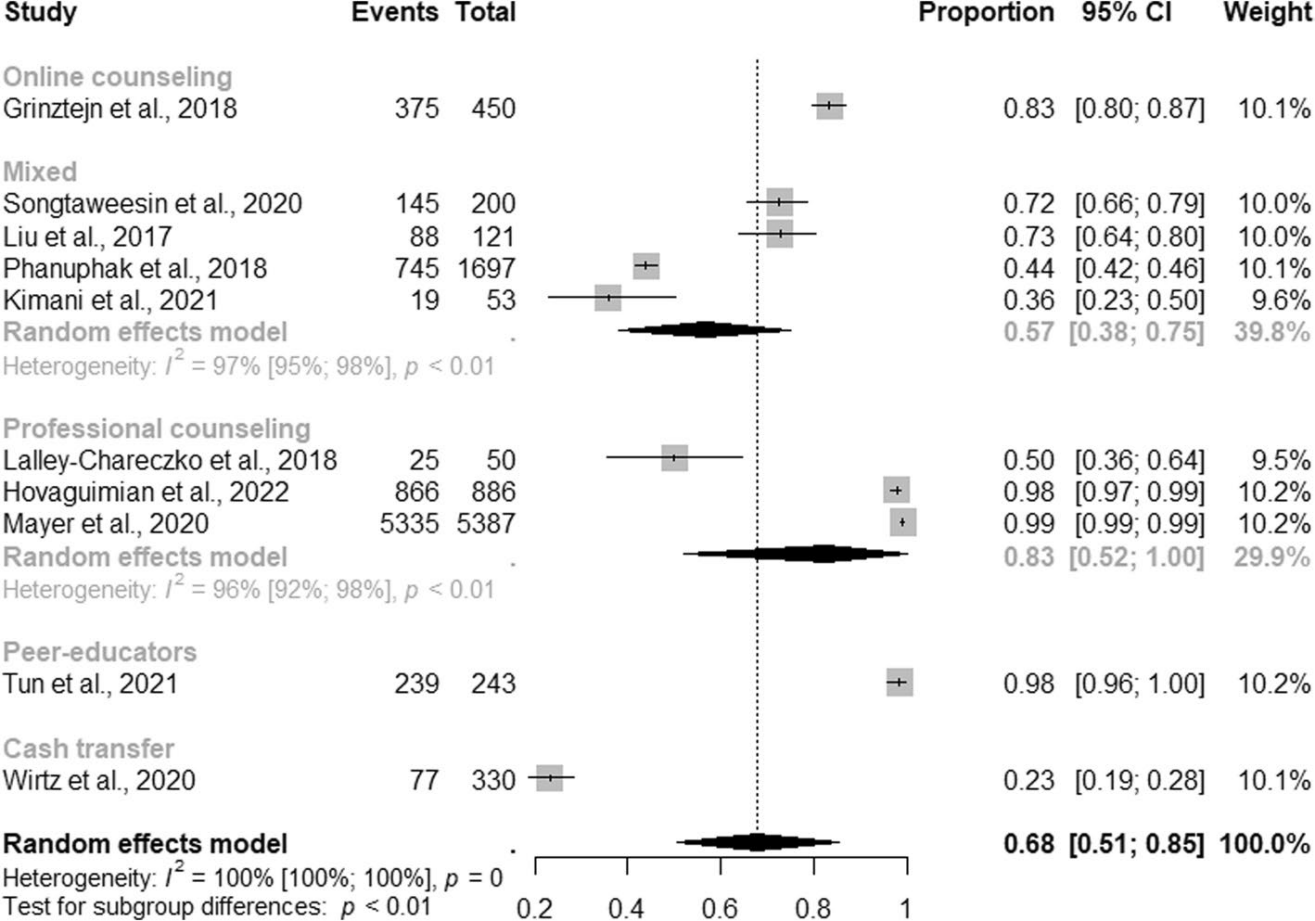
Forest plot of pooled proportions of retention to pre-exposure prophylaxis (PrEP) service among the entire sample (MSM and TGW) by retention strategies (*n*=10)

The subgroup analysis revealed a retention proportion of 57% [95% CI: 0.38–0.75] for mixed and 83% [95% CI: 0.52–1.00] for professional counseling (Fig. 8).

**Fig. 9:**
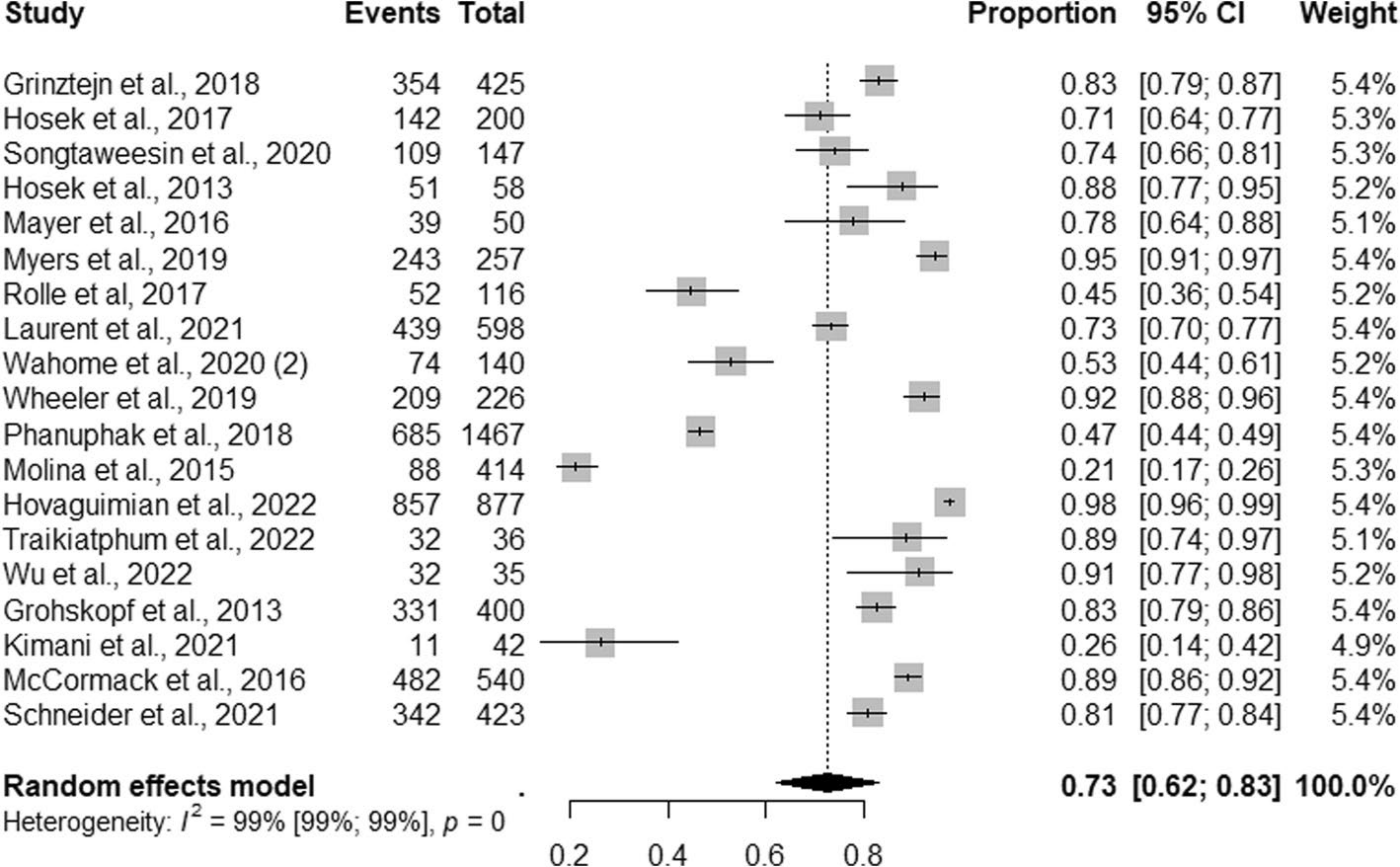
Forest plot of pooled proportions of retention to pre-exposure prophylaxis (PrEP) service among MSM (*n*=19)

The pooled proportions of retention to PrEP service among MSM was 73% (95% CI: 0.62–0.83; I^2^=100%) (Fig. 9).

**Fig. 10:**
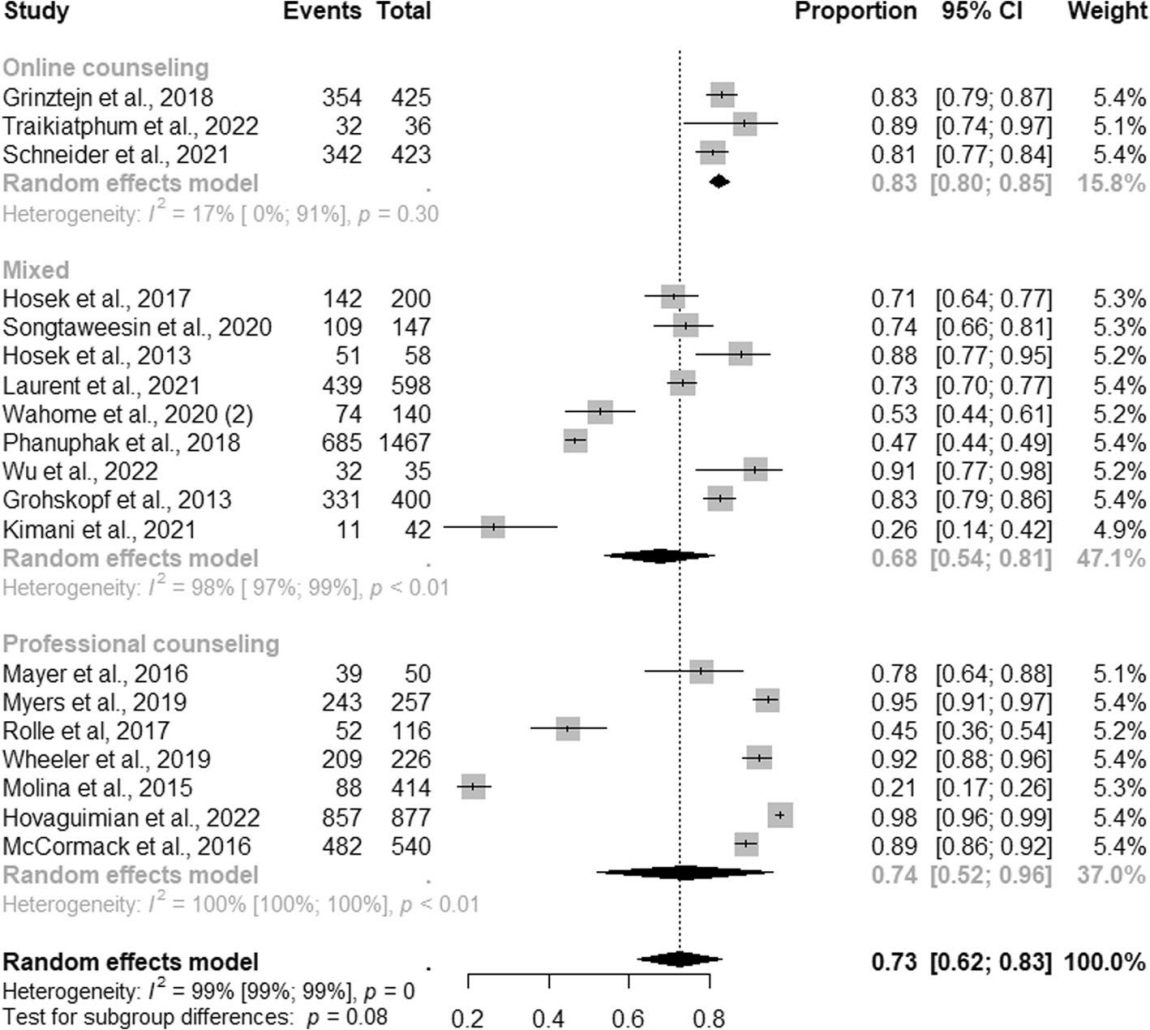
Forest plot of pooled proportions of retention to pre-exposure prophylaxis (PrEP) service among MSM by demand creation strategies (*n*=19)

Of these, 83% (95% CI: 0.80–0.85; I^2^=17%) were retained in PrEP provision services by online counseling; 68% (95% CI: 0.54–0.81; I ^2^=98%) by mixed and 74% 95% CI: 0.52–096; I^2^=100%) by professional counseling strategies (Fig. 10).

**Fig. 11:**
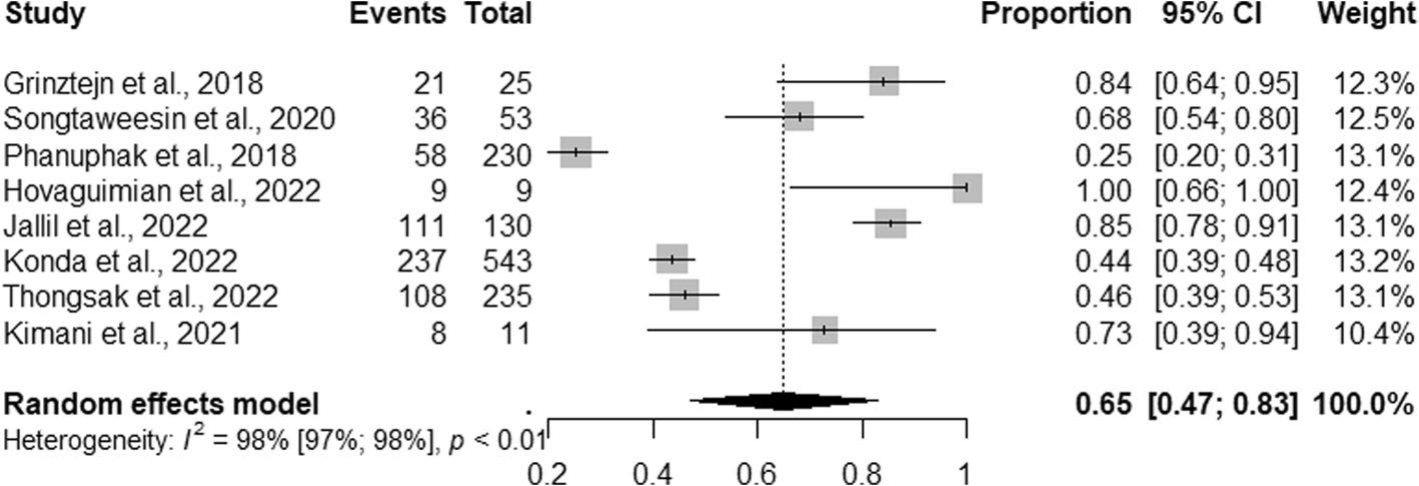
Forest plot of pooled proportions of retention to pre-exposure prophylaxis (PrEP) service among TGW (*n*=08)

Eighteen studies presented the retention data for TGW. The prevalence of retention to the PrEP service by TGW was 65% (95% CI: 0.47–0.83; I^2^=98%) (Fig. 11).

**Fig. 12:**
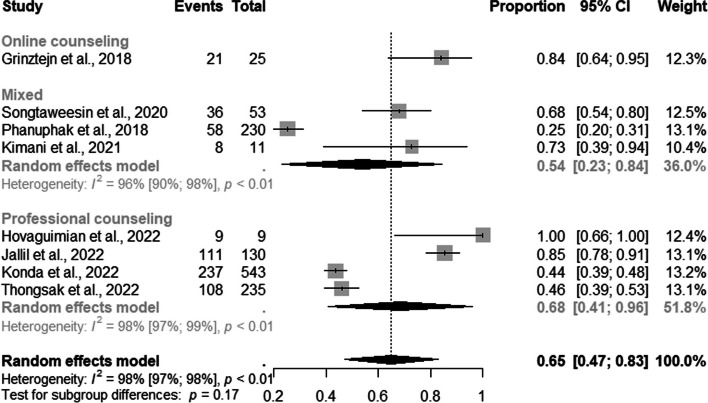
Forest plot of pooled proportions of retention to pre-exposure prophylaxis (PrEP) service among TGW by DCS (*n*=08)

In the subgroup analysis, we observed that 84% of the TGW were retained in PrEP provision services through online (95% CI: 0.64–0.95); 68% (95% CI: 0.41–0.96; I^2^=51.8%) through professional counseling, and 54% (95% CI: 0.23–0.84) through mixed strategies (Fig. 12).

### Meta-regression

In the meta-regression analysis, the studies were grouped according to study design (trial, cohort, cross-sectional), sample size (≤400; >400), study place (Asia; Western), setting (HIV prevention and care; population), monitoring (monthly; 2–3 months; 6 months), and risk of bias (low, moderate, and high) (Appendix 8). The covariates for the outcome of PrEP DCS did not differ significantly. However, there was a significant difference in the study design; the longer the study duration (cohort versus cross-sectional or RCT), the lower the proportions observed.

## Barriers and facilitators to PrEP retention

Of the 46 studies included, two described the barriers [37, 38], and three presented the facilitators [37, 62] (Grinztejn et al. 2018) to retaining this population in health services offering PrEP. Among the studies reporting barriers to retention in PrEP services, two focused on TGW [37, 38]. The barriers highlighted in these studies were social determinants of health [37],reporting condom-less anal sex (CAS) with partner(s) of unknown HIV status [38] and being an immigrant [38]. Studies reporting the facilitators for retaining this population in PrEP services include one study focused on TGW [37], and two on the total population (MSM and TGW) [44, 62], which highlighted facilitators, including PrEP offered at public health-care clinics in a middle-income setting [44], approach to counseling [62], multidisciplinary care [62], and gender-affirming settings [37].

## Discussion

Herein, we conducted a comprehensive search to identify DCS and RS with higher proportions among MSM and TGW to improve PrEP persistence, which is crucial for reducing the HIV epidemic. As a main result, online counseling had the highest proportions for DCS and RS. Meanwhile, mixed DCS and RS were the most frequent for TGW.

The COVID-19 pandemic has impacted the way interviews are conducted in the health area, causing a significant increase in the use of online approaches [73, 74]. Online research methodologies may serve as an important mechanism for population-focused data collection among young individual and have been acknowledged for their potential in investigating understudied and marginalized populations and subpopulations, permitting increased access to communities that tend to be less visible and, consequently, less studied in offline contexts [75, 76].

Online interviews present several advantages over face-to-face interviews, particularly when engaging with hard-to-reach populations such as MSM and TGW. These advantages include anonymity, instant access to services, peer-to-peer models of online outreach, and reduced barriers such as geography and time. However, online approaches require reading, technological literacy of participants, and access to technology, which may limit relationship-building between participants and researchers [77].

The COVID-19 pandemic has reinforced the need to diversify the strategies for recruiting and retaining in PrEP services [26] (Dourado et al. 2020). The pandemic context, which negatively impacts access of MSM and TGW to HIV testing and prevention services in multiple countries [78, 79], demonstrates the need to readapt strategies, aiming for more online resources due to the facilitation of communication between users and services through the use of various platforms, such as social networks, dating applications, and chatbots [26, 80].

Furthermore, although our systematic review and meta-analysis revealed an 84% DCS rate of PrEP use (95% CI: 77–91%), we observed a 62% retention rate of PrEP use (95% CI: 50–74%). HIV infection is disproportionally more frequent among MSM and TGW, and new infections are increasing in this population [81]. Therefore, PrEP is a critical prevention strategy among populations at substantial risk of HIV to reduce new infections [82]. According to RCTs results, once-daily and on-demand PrEP are effective among MSM and TGW. Nonetheless, adherence and retention to this therapy are significant challenges for effective PrEP implementation and are important determinants of the effectiveness of this pharmacotherapy in preventing HIV in clinical practice [44].

The PrEP DCS and RS were relatively similar among the populations studied. Specifically, the PrEP DCS was 92% (95% CI: 0.87–0.97) among MSM and 95% (95% CI: 0.84–1.00) among TGW; while the retention rate to PrEP service was 90% (95% CI: 0.84–0.96) and 91% (95% CI: 0.74–1.00) among MSM and TGW, respectively. The secondary outcome of this review was to assess the barriers to and facilitators of MSM and TGW retention in PrEP provision services. Four studies provided data on this outcome [37, 38, 44, 62]. Socioeconomic factors play an important role in retaining MSM and TGW in PrEP provision services. PrEP offered in public health clinics was a facilitator [44], which is an important finding, particularly in middle-income countries. A study with MSM conducted in the United States suggested that affordable PrEP and care were relevant factors for PrEP retention and continuum care [83].

Moreover, multidisciplinary care [62] and gender-affirming settings [37] appear to be facilitators, as corroborated by Rogers [83], who presented culturally tailored (LGBTQ+) clinical services as an alternative for enhancing PrEP persistence. In a qualitative study with transwomen in Brazil on barriers to and facilitators of PrEP, discrimination in the public health system (SUS) was identified as a barrier to PrEP, and misgendering was identified as a specific form of discrimination, reinforcing the findings of the studies included in this review [9]. Previous data indicate the importance of addressing the social determinants of health and economic barriers, such as the cost of PrEP medication and care, discrimination in health facilities, and the lack of multidisciplinary care. Alternative options include the provision of PrEP in public health services with a multidisciplinary care and the training of health care workers to provide gender-affirming care with sensibility.

This systematic review and meta-analysis had several strengths, including the availability of subgroup analyses by interview strategy and meta-regression to identify possible sources of heterogeneity. Nonetheless, some limitations should be considered. The risk of bias assessment showed that the main problems were related to the measurement of outcomes, participants, and study selection. Furthermore, high heterogeneity exists among the studies in the meta-analyses, which remained high after subgroup and meta-regression analyses. This high heterogeneity can be explained by differences in the study designs, selection bias in some studies, and differences in some population characteristics, such as age and educational level. Another potential limitation of our study is are the limited number of included studies focusing on the description of strategies for DCS on TGW and online strategy-isolating forms.

Raising PrEP awareness among MSM and TGW, minimizing gaps in access, and ensuring retention of PrEP services are critical issues. Offering PrEP through online DCS and RS can reach and retain high numbers of MSM and TGW, and reduce HIV incidence in these populations.

### Supplementary Information

**Additional file 1:** **Appendix 1.** Search strategy. **Appendix 2.** PRISMA flow-chart of this systematic review. **Appendix 3.** Excluded studies on full text lecture with respective reason for exclusion of search strategy (*N*=139). **Appendix 4.** Risk of bias recruitment of observational studies included studies at systematic review, 2022. **Appendix 5.** Risk of bias recruitment of interventional studies (trials) included studies at systematic review, 2022. **Appendix 6.** Risk of bias retention of observational studies included studies at systematic review, 2022. **Appendix 7.** Risk of bias retention of interventional studies (trials) included studies at systematic review, 2022. **Appendix 8.** Meta-regression according to selected covariates.

## Data Availability

All data generated or analyzed during this study are included in this published article [and its supplementary information files].
