# Supplementary material for: Demand creation and retention strategies for oral pre-exposure prophylaxis for HIV prevention among men who have sex with men and transgender women: a systematic review and meta-analysis
Source: BMC Infect Dis. 2023 Nov 14;23:793. doi: 10.1186/s12879-023-08693-z (PMC10644426; doi:10.1186/s12879-023-08693-z)
Supplement: Supplementary file 1 — Additional file 1: Appendix 1. Search strategy. Appendix 2. PRISMA flow-chart of this systematic review. Appendix 3. Excluded studies on full text lecture with respective reason for exclusion of search strategy (N=139). Appendix 4. Risk of bias recruitment of observational studies included studies at systematic review, 2022. Appendix 5. Risk of bias recruitment of interventional studies (trials) included studies at systematic review, 2022. Appendix 6. Risk of bias retention of observational studies included studies at systematic review, 2022. Appendix 7. Risk of bias retention of interventional studies (trials) included studies at systematic review, 2022. Appendix 8. Meta-regression according to selected covariates. [file 12879_2023_8693_MOESM1_ESM.docx]

**Appendix 1 – Search strategy**

# 1. Research Algorithms

Following there are search strategies in different databases (PubMed, Embase, Web of Science, Cochrane Library and Biblioteca Virtual em Saúde – LILACS, IBECS,  PAHOIRIS, WHOLIS)

1. **Search Strategy for PubMed/Medline**

#1 "Pre-Exposure Prophylaxis" [MeSH Terms] OR (Pre Exposure Prophylaxis) OR (Pre-Exposure Prophylaxi) OR (Prophylaxi, Pre-Exposure) OR (Prophylaxis, Pre-Exposure) OR (Pre-Exposure Prophylaxis (PrEP)) OR (Pre Exposure Prophylaxis (PrEP)) OR (Pre-Exposure Prophylaxi (PrEP)) OR (Prophylaxi, Pre-Exposure (PrEP)) OR (Prophylaxis, Pre-Exposure (PrEP))

#2 "Homosexuality, Male" [MeSH Terms] OR (Male Homosexuality) OR "Transgender Persons" [MeSH Terms] OR (Person, Transgender) OR (Transgender Person) OR (Transgenders) OR (Transgender) OR (Transgendered Persons) OR (Person, Transgendered) OR (Persons, Transgendered) OR (Transgendered Person) OR (Two-Spirit Persons) OR (Person, Two-Spirit) OR (Two Spirit Persons) OR (Two-Spirit Person) OR (Transsexual Persons) OR (Person, Transsexual) OR (Transsexual Person) OR (Transexuals) OR (Transexual) OR "Sexual and Gender Minorities" [MeSH Terms] OR (LGBT Persons) OR (LGBT Person) OR (Person, LGBT) OR (Persons, LGBT) OR (LGBTQ Persons) OR (LGBTQ Person) OR (Person, LGBTQ) OR (Persons, LGBTQ) OR (Non-Heterosexual Persons) OR (Non Heterosexual Persons) OR (Non-Heterosexual Person) OR (Person, Non-Heterosexual) OR (LBG Persons) OR (LBG Person) OR (Person, LBG) OR (Persons, LBG) OR (Sexual Minorities) OR (Minorities, Sexual) OR (Minority, Sexual) OR (Sexual Minority) OR (Non-Heterosexuals) OR (Non Heterosexuals) OR (Non-Heterosexual) OR (GLBT Persons) OR (GLBT Person) OR (Person, GLBT) OR (Persons, GLBT) OR (GLBTQ Persons) OR (GLBTQ Person) OR (Person, GLBTQ) OR (Persons, GLBTQ) OR (Gays) OR (Gay) OR (Men Who Have Sex With Men) OR (Gender Minorities) OR (Gender Minority) OR (Minorities, Gender) OR (Minority, Gender)  OR (Homosexuals) OR (Homosexual)

#3 Adolescent [MeSH Terms] OR (Adolescents) OR (Adolescence) OR (Teens) OR (Teen) OR (Teenagers) OR (Teenager) OR (Youth) OR (Youths) OR (Adolescents, Female) OR (Adolescent, Female) OR (Female Adolescent) OR (Female Adolescents) OR (Adolescents, Male) OR (Adolescent, Male) OR (Male Adolescent) OR (Male Adolescents) OR **Young Adult** [MeSH Terms] OR  (Adult, Young) OR (Adults, Young) OR (Young Adults)

"cohort studies"[mesh] OR "case-control studies"[mesh] OR "comparative study"[pt] OR "risk factors"[mesh] OR "cohort"[tw] OR "compared"[tw] OR "groups"[tw] OR "case control"[tw] OR "multivariate"[tw]

**Identification of studies via other methods**

**Identification of studies via databases**

**Identification**

Records identified from:

Citation searching (n = 07)

Records identified from*:

Databases (n = 1.129)

Records removed *before screening*:

Records marked as ineligible by automation tools (n =213)

Reports sought for retrieval

(n = 169)

Records screened

(N=916 n=782 Rayyan + 134 Cochrane and LILACS)

Records excluded**

(n = 747)

**Screening**

Reports assessed for eligibility

(n = 7)

Reports excluded (n= 139)

Measures the participants' intention to use (n=32) Retrospective study (n=06) Secondary study (n=01) Not measure outcomes (n=57) Mixed population (n=12) Non-specific population (n=06) Qualitative study (n=08) Comment (n=01)

Language (n=01)

Protocol without results (n=01)

Previous PrEP use (n=01)

Without actually using (n=06)

Review (n=01)

Duplicate (n=01)

Modeling (n=01)

No recruiting strategy (n=04)

**Included**

Studies included in review via database (n = 30)

Studies included in review via other methods (n =07)

Uptade (n=09)

Total included studies (n = 46)

**Appendix 2** – PRISMA flow-chart of this systematic review.

**Appendix 3 - Excluded studies on full text lecture with respective reason for exclusion of search strategy (N=139)**

| **Title** | **Reference** | **Exclusion** |
| --- | --- | --- |
| Kidney function and daily emtricitabine/tenofovir disoproxil fumarate pre-exposure prophylaxis against HIV: results from the real-life multicentric demonstrative project PrEP Brazil | Petruccelli et al., 2022  AIDS Res Ther | retrospective analysis |
| iPrevent: Engaging youth as long-acting HIV prevention product co-researchers in Cape Town, South Africa | Hartmann et al., 2021  Afr. J. AIDS Res. | not PrEP use |
| Pre-exposure prophylaxis persistence is a critical issue in prep implementation | Spinelli et al., 2020  Clin. Infect. Dis. | Comment |
| Prep in student health services: An opportunity to expand | Rawlins-Pilgrim et al., 2020  J. Gen. Intern. Med. | not PrEP use |
| Neighbourhoods, networks and pre-exposure prophylaxis awareness: A multilevel analysis of a sample of young black men who have sex with men | Chen et al., 2019  Sex. Transm. Infect. | PrEP awareness |
| Estimated prevalence of adolescents and young adults with indications for hiv pre-exposure prophylaxis in the united states, 2011-2015 | Allen et al., 2018  J. Adolesc. Health | Non-specific population: adolescents and young adults (AYA) |
| Implementing HIV pre-exposure prophylaxis education and management strategies for providers in an STI/HIV screening program within an adolescent/young adult practice | Einhorn et al., 2018  J. Adolesc. Health | Non-specific population: :adolescents and young adults (AYA) |
| Acceptability of Oral Preexposure Prophylaxis among Men Who Have Sex with Men in Philadelphia | Adams et al., 2016  J. Acquired Immune Defic. Syndr. | not PrEP use |
| I am men's health: Generating adherence to HIV pre-exposure prophylaxis (prep) in young men of color who have sex with men | Daughtridge et al., 2015  J. Int. Assoc. Providers AIDS Care | without outcome (adherence) |
| Syphilis predicts HIV incidence among men and transgender women who have sex with men in a preexposure prophylaxis trial | Solomon et al., 2014  Clin. Infect. Dis. | without outcome (HIV incidence) |
| Factors influencing uptake, continuation, and discontinuation of oral PrEP among clients at sex worker and MSM facilities in South Africa. | Pillay et al., 2020  PloS One | Non-specific population: elderly |
| Uptake of PrEP and condom and sexual risk behavior among MSM during the ANRS IPERGAY trial. | Sagaon-Teyssier et al., 2016  AIDS Care | without outcome (sexual risk behavior) |
| HIV sero disclosure among men who have sex with men and transgender women on HIV pre-exposure prophylaxis. | Hojilla et al., 2018  AIDS Care | without outcome (lack of knowledge) and other subjects (partner) |
| Breaking Inertia: Movement Along the PrEP Cascade in a Longitudinal US National Cohort of Sexual Minority Individuals at Risk for HIV. | Mehrotra et al., 2021  Journal of Acquired Immune Deficiency Syndromes | Mixed population - transmale |
| Awareness of and willingness to use pre-exposure prophylaxis (PrEP) among people who inject drugs and men who have sex with men in India: Results from a multi-city cross-sectional survey. | Belludi et al., 2021  PloS One | without outcome (awareness) |
| Predictors of Over-Reporting HIV Pre-exposure Prophylaxis (PrEP) Adherence Among Young Men Who Have Sex With Men (YMSM) in Self-Reported Versus Biomarker Data. | Baker et al., 2018  AIDS and Behavior | without outcome (adherence) |
| Safety and adherence to intermittent pre-exposure prophylaxis (PrEP) for HIV-1 in African men who have sex with men and female sex workers. | Mutua et al., 2012  PloS One | Mixed population |
| Enhancing PrEP Access for Black and Latino Men Who Have Sex With Men. | Lelutiu-Weinberger et al., 2016  Journal of Acquired Immune Deficiency Syndromes | not PrEP use (perceived barriers and facilitators to PrEP access) |
| Knowledge, Indications and Willingness to Take Pre-Exposure Prophylaxis among Transwomen in San Francisco, 2013. | Wilson et al., 2015  PloS One | without outcome (knowledge, indications and willingness ) |
| Demographic, Behavioral, and Geographic Differences Between Men, Transmen, and Transwomen Currently on PrEP, Former PrEP Users, and Those Having Never Used PrEP. | Westmoreland et al., 2020  AIDS and Behavior | not PrEP use and transmale |
| PrEP Use and Adherence Among Transgender Patients | Downing et al., 2022  AIDS and Behavior | Mixed population (transgender and cisgender men and women) |
| Engagement in the pre-exposure prophylaxis (PrEP) cascade among a respondent-driven sample of sexually active men who have sex with men and transgender women during early PrEP implementation in Zimbabwe | Parmley et al., 2022  J. Int. AIDS Soc. | without outcome (awareness) |
| Living while black, gay, and poor: The association of race, neighborhood structural disadvantage, and prep utilization among a sample of black men who have sex with men in the deep south | Burns et al., 2021  AIDS Educ. Prev. | not PrEP use |
| TFV-DP AND FTC-TP in pbmc among transgender adolescents receiving daily TDF/FTC | Yager et al., 2021  Top. Antiviral Med. | not PrEP use |
| Baseline demographics, coverage and first regimen choice of participants in the HIV Pre-Exposure Prophylaxis (PrEP) Impact trial | Sullivan et al., 2021  J. Int. AIDS Soc. | retrospective analysis |
| Exposure to Truvada class-action lawsuit advertisements among young men who have sex with men and transgender women in Chicago, Illinois, USA | Macapagal et al., 2021  J. Int. AIDS Soc. | without outcome (awareness) |
| Prevalence of syphilis among adolescents' men who have sex with men (MSM) and transgender women (TGW) in Brazil | Gomes et al., 2021  J. Int. AIDS Soc. | without outcome (syphilis prevalence) |
| Null effect of financial incentives or social media support on prep adherence in a randomized controlled trial of young men who have sex with men of colour | Ware et al., 2020  Open Forum Infect. Dis. | without outcome (awareness) |
| PrEPTECH - Integration of an on- and off-line holistic PrEP solution for youth | Braun et al., 2020  J. Int. AIDS Soc. | qualitative study |
| Lynx: A pilot randomized controlled trial of a mobile health HIV testing and PrEP uptake intervention for young men who have sex with men | Scott et al., 2020  J. Int. AIDS Soc. | without outcome (app implementation) |
| Toward a better understanding of PrEP-related behavior: PrEP awareness, past and current use, and intent to use PrEP in the future among HIV-negative MSM in three US cities, 2018 | Mansergh et al., 2019  Ann. Epidemiol. | without outcome (awareness) |
| PrEP implementation among MSM in Ukraine: Results of pilot project in Kyiv | Marchenko et al., 2019  HIV Med. | protocol without results |
| Close group social media network and social gatherings for comprehensive HIV services among adolescent MSM in Lagos Nigeria | Enadeghe et al., 2019  HIV Med. | qualitative study |
| Utilization and avoidance of sexual health services and providers by YMSM and transgender youth assigned male at birth in Chicago | Phillips et al., 2019  AIDS Care Psychol. Socio-Med. Asp. AIDS HIV | without outcome (use or avoid sexual health services) |
| Prep acceptability, uptake, and adherence among young men who have sex with men and transgender women in PrEP demonstration project, Chiang Mai, Thailand | Kaewpoowat et al., 2019  Open Forum Infect. Dis. | no recruiting strategy |
| HPTN 067/ADAPT: Correlates of Sex-Related Pre-exposure Prophylaxis Adherence, Thai Men Who Have Sex with Men, and Transgender Women, 2012-2013 | Holtz et al., 2019  J. Acquired Immune Defic. Syndr. | without outcome (adherence) |
| Predictors of PrEP Uptake Among Patients with Equivalent Access | Golub et al., 2019  AIDS Behav | without outcome (adherence) |
| Developing a novel mobile app to support HIV testing and PrEP uptake among young MSM: The LYNX Study | Liu et al., 2019  J. Int. AIDS Soc. | qualitative study |
| Are we enrolling men who have sex with men with greatest HIV acquisition risk in a preexposure prophylaxis program? | Fields et al., 2019  Sex. Transm. Infect. | without outcome (HIV risk factors) |
| Prep utilization among young transgender women, transgender men, and msm in an urban community-based setting | Dankerlui et al., 2019  Sex. Transm. Infect. | without outcome (adherence) |
| Unmet material and social needs and pre-exposure prophylaxis (PREP) for HIV prevention adoption: What is the association? | Andriano et al., 2019  J. Gen. Intern. Med. | not PrEP use and transmale |
| Influencing Factors of Pre-Exposure Prophylaxis Self-Efficacy Among Men Who Have Sex With Men | Qu et al., 2019  Am J Mens Health | without outcome (adherence) |
| Characteristics of Social Support Networks Among Young Men And Transgender Women of Color Receiving Pre-Exposure Prophylaxis (PrEP) For HIV Prevention | Wood et al., 2019  J. Adolesc. Health | qualitative study |
| PrEP uptake preferences among men who have sex with men in China: results from a National Internet Survey | Han et al., 2019  J. Int. AIDS Soc. | without outcome (awareness) |
| Considerable interest in pre-exposure prophylaxis uptake among men who have sex with men recruited from a popular geosocial-networking smartphone application in London | Goedel et al., 2019  Global Public Health | without outcome (awareness) |
| Influence of prep4love campaign on prep uptake among ymsm in Chicago | Phillips et al., 2019  Top. Antiviral Med. | without actually using |
| Prep-related barriers among men who have sex with men in Brazil, Mexico, & Peru | Ofori et al., 2019  Top. Antiviral Med. | without outcome (awareness) |
| Linkage to care in the partner services pre-exposure prophylaxis (ps-prep) study | Da Silva et al., 2019  Top. Antiviral Med. | not PrEP use |
| Pilot test of a prep telemedicine system for young black MSM in the rural us south | Siegler et al., 2019  Top. Antiviral Med. | not PrEP use (PrEP prescription filled and acceptability) |
| Threefold Increase in PrEP Uptake Over Time with High Adherence Among Young Men Who Have Sex With Men in Chicago | Morgan et al., 2018  AIDS and Behavior | without actually using |
| Awareness, knowledge, use, willingness to use and need of Pre-Exposure Prophylaxis (PrEP) during World Gay Pride 2017 | Iniesta et al., 2018  PLoS One | Mixed population - transmale |
| Beyond the Biomedical: Preexposure Prophylaxis Failures in a Cohort of Young Black Men Who Have Sex with Men in Atlanta, Georgia | Serota et al., 2018  Clin. Infect. Dis. | review |
| P3 (Prepared, Protected, emPowered): Feasibility and acceptability of a PrEP adherence app featuring peer-topeer sharing, game-based elements and in-app adherence counseling | Legrand et al., 2018  J. Int. AIDS Soc. | not PrEP use (feasibility and acceptability) |
| Daily and Nondaily Oral Preexposure Prophylaxis in Men and Transgender Women Who Have Sex with Men: The Human Immunodeficiency Virus Prevention Trials Network 067/ADAPT Study | Grant et al., 2018  Clin. Infect. Dis. | without outcome (adherence) |
| Trends in PrEP uptake, adherence, and discontinuation among YMSM in Chicago | Morgan et al., 2018  Top. Antiviral Med. | without outcome (adherence) |
| Acceptability of native mobile phone applications (apps) in sexual health: Is an app acceptable in men who have sex with men for those taking or considering taking PrEP | Bayley et al., 2018  HIV Med. | without outcome (app adherence) |
| The trends in PrEP awareness, acceptability and uptake amongst MSM in London between 2016 and 2018 | Bayley et al., 2018  HIV Med. | without outcome (awareness) |
| Demand for pre-exposure prophylaxis for HIV and the impact on clinical services: Scottish men who have sex with men perspectives | Gilson et al., 2018  Int. J. STD AIDS | without outcome (willingness) |
| Comparison of Measures of Adherence to Human Immunodeficiency Virus Preexposure Prophylaxis among Adolescent and Young Men Who Have Sex with Men in the United States | Koss et al., 2018  Clin. Infect. Dis. | without outcome (adherence) |
| Choosing between daily and event-driven pre-exposure prophylaxis: Results of a Belgian PrEP demonstration project | Reyniers et al., 2018  J. Acquired Immune Defic. Syndr. | without outcome (sexual risk-taking) |
| Awareness, willingness, and PrEP eligibility among transgender women in Rio de Janeiro, Brazil | Jalil et al., 2018  J. Acquired Immune Defic. Syndr. | without outcome (awareness, willingness) |
| Correlates of Preexposure Prophylaxis (PrEP) Use among Men Who Have Sex with Men (MSM) in Los Angeles, California | Okafor et al., 2017  J Urban Health | without actually using |
| Client-centered counseling-based resource center increased uptake of HIV pre-exposure prophylaxis (PREP) in a randomized controlled trial of young black men who have sex with men | Desrosiers et al., 2017  Open Forum Infect. Dis. | no recruiting strategy |
| It's just not for me: Exploring low prep uptake among young black men who have sex with men in the Southern United States | Pingel et al., 2017  Sex. Transm. Infect. | qualitative study |
| Findings from the men who have sex with men (MSM) internet survey ireland (MISI): Estimated proportion of MISI respondents eligible for pre-exposure prophylaxis (PrEP) | Nic Lochlainn et al., 2017  Sex. Transm. Infect. | without actually using |
| Text messaging is associated with improved retention in a clinic-based PrEP program | Khosropour et al., 2017  Top. Antiviral Med. | not PrEP use |
| Prevalence and characteristics of users of pre-exposure prophylaxis (PrEP) among men who have sex with men, San Francisco, 2014 in a cross-sectional survey: Implications for disparities | Snowden et al., 2017  Sex. Transm. Infect. | not PrEP use (access to PrEP) |
| Brief Report: PrEP Uptake, Adherence, and Discontinuation among California YMSM Using Geosocial Networking Applications | Holloway et al., 2017  J. Acquired Immune Defic. Syndr. | not PrEP use |
| Who will use pre-exposure prophylaxis (PrEP) and why?: Understanding PrEP awareness and acceptability amongst men who have sex with men in the UK - A mixed methods study | Frankis et al., 2016  PLoS One | qualitative study |
| Antiretroviral pre-exposure prophylaxis preferences among men who have sex with men in Vietnam: Results from a nationwide cross-sectional survey | Oldenburg et al., 2016  Sex. Health | not PrEP use (preferences) |
| Factors associated with self-disclosure of sexual behavior to healthcare providers amongst young men who have sex with men in Washington DC | Levy et al., 2016  AIDS Res. Hum. Retroviruses | not PrEP use (preferences) |
| Long-term safety and efficacy of emtricitabine and tenofovir alafenamide vs emtricitabine and tenofovir disoproxil fumarate for HIV-1 pre-exposure prophylaxis: week 96 results from a randomised, double-blind, placebo-controlled, phase 3 trial. | Ogbuagu et al., 2021  The Lancet HIV | not PrEP use (safety) |
| Uptake of pre-exposure prophylaxis, sexual practices, and HIV incidence in men and transgender women who have sex with men: a cohort study. | Grant et al., 2014  The Lancet Infectious Diseases | secondary analysis |
| Pre-exposure prophylaxis among men who have sex with men in Côte d'Ivoire: a quantitative study of acceptability. | Diabaté et al., 2021  AIDS Care | without outcome (awareness) |
| Decentralizing PrEP delivery: Implementation and dissemination strategies to increase PrEP uptake among MSM in Toronto, Canada. | Charest et al., 2021  PloS One | without actually using |
| The PrEP Cascade in a National Cohort of Adolescent Men Who Have Sex With Men. | Moskowitz et al., 2021  Journal of Acquired Immune Deficiency Syndromes | without outcome |
| Study on Pre-Exposure Prophylaxis Regimens among Men Who Have Sex with Men: A Prospective Cohort Study. | Wu et al., 2019  International Journal of Environmental Research and Public Health | without outcome (adherence) |
| Role of Social and Sexual Network Factors in PrEP Utilization Among YMSM and Transgender Women in Chicago. | Phillips et al., 2019  Prevention Science: the Official Journal of the Society for Prevention Research | without outcome (factors associated with HIV infection) |
| Engagement of Latino immigrant men who have sex with men for HIV prevention through eHealth: preferences across social media platforms. | Lee et al., 2021  Ethnicity & Health | qualitative study |
| PrEP4Love: The Role of Messaging and Prevention Advocacy in PrEP Attitudes, Perceptions, and Uptake Among YMSM and Transgender Women. | Phillips et al., 2019  Journal of Acquired Immune Deficiency Syndromes (1999) | not PrEP use |
| HIV testing and pre-exposure prophylaxis (PrEP) use, familiarity, and attitudes among gay and bisexual men in the United States: A national probability sample of three birth cohorts. | Hammack et al., 2018  PloS One | no recruiting strategy |
| The Role of Social Support in HIV Testing and PrEP Awareness among Young Black Men and Transgender Women Who Have Sex with Men or Transgender Women. | Lelutiu-Weinberger et al., 2020  Journal of Urban Health | without outcome (awareness) |
| Preexposure Prophylaxis for HIV Infection Integrated With Municipal- and Community-Based Sexual Health Services. | Liu et al., 2016  JAMA Internal Medicine | without outcome (PrEP willingness) |
| Imperfect adherence in real life: a prevention-effective perspective on adherence to daily and event-driven HIV pre-exposure prophylaxis among men who have sex with men - a prospective cohort study in Taiwan. | Wu et al., 2021  Journal of the International AIDS Society | without outcome (awareness) |
| Understanding the impact of a syndemic on the use of pre-exposure prophylaxis in a community-based sample of behaviorally PrEP-eligible BMSM in the United States. | Chandler et al., 2020  AIDS Care | not PrEP use |
| HIV Prevalence and Associated Risk Factors Among Men Who Have Sex With Men in Dar es Salaam, Tanzania. | Mmbaga et al., 2018  Journal of Acquired Immune Deficiency Syndromes (1999) | without outcome |
| Social-Environmental Resilience, PrEP Uptake, and Viral Suppression among Young Black Men Who Have Sex with Men and Young Black Transgender Women: the Neighborhoods and Networks (N2) Study in Chicago. | Chen et al., 2020  Journal of Urban Health | not PrEP use |
| Brief Report: Associations Between Self-Reported Substance Use Behaviors and PrEP Acceptance and Adherence Among Black MSM in the HPTN 073 Study. | Okafor et al., 2020  Journal of Acquired Immune Deficiency Syndromes (1999) | without outcome (adherence) |
| Population-Level Sexual Mixing According to HIV Status and Preexposure Prophylaxis Use Among Men Who Have Sex With Men in Montreal, Canada: Implications for HIV Prevention. | Wang et al., 2020  American Journal of Epidemiology | without outcome |
| Potential Impact of Interventions to Enhance Retention in Care During Real-World HIV Pre-Exposure Prophylaxis Implementation. | Chan et al., 2019  AIDS Patient Care and STDs | without outcome |
| Quarterly screening optimizes detection of sexually transmitted infections when prescribing HIV preexposure prophylaxis. | Tang et al., 2020  AIDS (London, England) | without outcome (STI) |
| Barriers to preexposure prophylaxis use among individuals with recently acquired HIV infection in Northern California. | Marcus et al., 2019  AIDS Care | without outcome (awareness) |
| Pre-exposure prophylaxis: awareness, acceptability and risk compensation behaviour among men who have sex with men and the transgender population. | Uthappa et al., 2018  HIV Medicine | not PrEP use |
| National trends in HIV pre-exposure prophylaxis awareness, willingness and use among United States men who have sex with men recruited online, 2013 through 2017. | Sullivan et al., 2020  Journal of the International AIDS Society | retrospective analysis |
| Estimated Impact of Targeted Pre-Exposure Prophylaxis: Strategies for Men Who Have Sex with Men in the United States. | Elion et al., 2019  International Journal of Environmental Research and Public Health | modeling study |
| Brief Report: PrEPTECH: A Telehealth-Based Initiation Program for HIV Pre-exposure Prophylaxis in Young Men of Color Who Have Sex With Men. A Pilot Study of Feasibility. | Refugio et al., 2019  Journal of Acquired Immune Deficiency Syndromes (1999) | without outcome (PrEP awareness, willingness) |
| Facebook network structure and awareness of preexposure prophylaxis among young men who have sex with men. | Khanna et al., 2017  Annals of Epidemiology | without outcome (awareness) |
| Use of pre-exposure prophylaxis increases the odds of condomless anal sex among young men who have sex with men (MSM) of colour. | Crosby et al., 2020  Sexual Health | without outcome (safety) |
| A Mobile Health Strategy to Support Adherence to Antiretroviral Preexposure Prophylaxis. | Fuchs et al., 2018  AIDS Patient Care and STDs | no recruiting strategy |
| High risk and low uptake of pre-exposure prophylaxis to prevent HIV acquisition in a national online sample of transgender men who have sex with men in the United States. | Reisner et al., 2019  Journal of the International AIDS Society | without outcome (awareness) |
| Pre-Exposure Prophylaxis (PrEP) Use and Condomless Anal Sex: Evidence of Risk Compensation in a Cohort of Young Men Who Have Sex with Men. | Newcomb et al., 2018  Journal of Acquired Immune Deficiency Syndromes (1999) | duplicate |
| Prevalence of actual uptake and willingness to use pre-exposure prophylaxis to prevent HIV acquisition among men who have sex with men in Hong Kong, China. | Wang et al., 2018  PloS One | without outcome (willingness) |
| Daily and event-driven pre-exposure prophylaxis for men who have sex with men in Belgium: results of a prospective cohort measuring adherence, sexual behaviour and STI incidence. | Vuylsteke et al., 2019  Journal of the International AIDS Society | without outcome (adherence) |
| Patterns and correlates of PrEP drug detection among MSM and transgender women in the Global iPrEx Study. | Liu et al., 2014  Journal of Acquired Immune Deficiency Syndromes (1999) | without outcome (adherence) |
| HIV pre-exposure prophylaxis in men who have sex with men and transgender women: a secondary analysis of a phase 3 randomised controlled efficacy trial. | Buchbinder et al., 2014  The Lancet Infectious Diseases | retrospective analysis |
| Disparities of HIV risk and PrEP use among transgender women of color in South Florida. | Holder et al., 2019  Journal of the National Medical Association | without outcome (awareness) |
| The Social Context of HIV Prevention and Care among Black Men Who Have Sex with Men in Three U.S. Cities: The Neighborhoods and Networks (N2) Cohort Study. | Duncan et al., 2019  International Journal of Environmental Research and Public Health | Mixed population |
| HIV incidence after pre-exposure prophylaxis initiation among women and men at elevated HIV risk: A population-based study in rural Kenya and Uganda. | Koss et al., 2021  PLoS Medicine | Mixed population |
| Perceived likelihood of using HIV pre-exposure prophylaxis medications among young men who have sex with men. | Mustanski et al., 2013  AIDS and Behavior | not PrEP use |
| Pre-exposure Prophylaxis Use and Detected Sexually Transmitted Infections Among Men Who Have Sex With Men in the United States-National HIV Behavioral Surveillance, 5 US Cities, 2017. | Chapin-Bardales et al., 2020  Journal of Acquired Immune Deficiency Syndromes (1999) | without actually using |
| From efficacy to effectiveness: facilitators and barriers to PrEP acceptability and motivations for adherence among MSM and transgender women in New York City. | Golub et al., 2013  AIDS Patient Care and STDs | not PrEP use |
| Healthcare Access and PrEP Continuation in San Francisco and Miami After the US PrEP Demo Project. | Doblecki-Lewis et al., 2017  Journal of Acquired Immune Deficiency Syndromes (1999) | retrospective analysis |
| Factors associated with reporting antibiotic use as STI prophylaxis among HIV PrEP users: findings from a cross-sectional online community survey, May-July 2019, UK. | O'Halloran et al., 2021  Sexually Transmitted Infections | previous PrEP use |
| Minimal Awareness and Stalled Uptake of Pre-Exposure Prophylaxis (PrEP) Among at Risk, HIV-Negative, Black Men Who Have Sex with Men. | Eaton et al., 2015  AIDS Patient Care and STDs | without outcome (awareness) |
| Intimacy motivations and pre-exposure prophylaxis (PrEP) adoption intentions among HIV-negative men who have sex with men (MSM) in romantic relationships. | Gamarel et al., 2015  Annals of Behavioral Medicine | not PrEP use |
| PrEP Use and Sexually Transmitted Infections Are Not Associated Longitudinally in a Cohort Study of Young Men Who Have Sex with Men and Transgender Women in Chicago. | Morgan et al., 2020  AIDS and Behavior | without outcome (STI) |
| [HIV self-testing reagent use in pre-exposure prophylaxis and related factors in men who have sex with men]. | Jin et al., 2021  Zhonghua liu xing bing xue za zhi | Language (japonese) |
| Outcomes of Preexposure Prophylaxis Referrals From Public STI Clinics and Implications for the Preexposure Prophylaxis Continuum. | Bhatia et al., 2018  Sexually Transmitted Diseases | without outcome |
| Pre-exposure Prophylaxis (PrEP) Use, Seroadaptation, and Sexual Behavior Among Men Who Have Sex with Men, San Francisco, 2004-2014. | Chen et al., 2016  AIDS and Behavior | not PrEP use |
| A novel Online-to-Offline (O2O) model for pre-exposure prophylaxis and HIV testing scale up. | Anand et al., 2017  Journal of the International AIDS Society | not PrEP use |
| Exploration of the Complex Relationships Among Multilevel Predictors of PrEP Use Among Men Who Have Sex with Men in the United States. | Rodriguez et al., 2021  AIDS and Behavior | not PrEP use |
| Associations between perceived barriers and benefits of using HIV pre-exposure prophylaxis and medication adherence among men who have sex with men in Western China. | Hu et al., 2018  BMC Infectious Diseases | without outcome |
| A Randomized Controlled Pilot Study of a Culturally-Tailored Counseling Intervention to Increase Uptake of HIV Pre-exposure Prophylaxis Among Young Black Men Who Have Sex with Men in Washington, DC. | Desrosiers et al., 2019  AIDS and Behavior | without outcome (willingness) |
| I am not a man': Trans-specific barriers and facilitators to PrEP acceptability among transgender women. | Sevelius et al., 2016  Global Public Health | qualitative study |
| Correlates of nonadherence to key population-led HIV pre-exposure prophylaxis services among Thai men who have sex with men and transgender women. | Seekaew et al., 2019  BMC Public Health | without outcome (adherence) |
| HIV Preexposure Prophylaxis Initiation at a Large Community Clinic: Differences Between Eligibility, Awareness, and Uptake. | Shover et al., 2018  American Journal of Public Health | mixed population |
| The Role of Social Relationships in PrEP Uptake and Use Among Transgender Women and Men Who Have Sex with Men. | Mehrotra et al., 2018  AIDS and Behavior | other population |
| Acceptability of Antiretroviral Pre-exposure Prophylaxis from a Cohort of Sexually Experienced Young Transgender Women in Two US Cities | Restar et al., 2018  AIDS and Behavior | not PrEP use |
| Characterizing the HIV Prevention and Care Continua in a Sample of Transgender Youth in the US | Reisner et al., 2017  AIDS and Behavior | not PrEP use |
| The "Safe Sex" Conundrum: Anticipated Stigma From Sexual Partners as a Barrier to PrEP Use Among Substance Using MSM Engaging in Transactional Sex. | Biello et al., 2017  AIDS and Behavior | not PrEP use |
| Factors Associated with Retention and Adherence in a Comprehensive, Diverse HIV Pre-Exposure Prophylaxis Clinic. | Goodman et al., 2022  AIDS Research and Human Retroviruses | retrospective analysis |
| Sexually transmitted infections incidence in young Thai men who have sex with men and transgender women using HIV pre-exposure prophylaxis. | Songtaweesin et al., 2022  International Journal of STD & AIDS | previous PrEP use |
| Longitudinal predictors of prep discontinuation among ymsm and transgender women | Newcomb et al., 2019  Top. Antiviral Med. | no recruiting strategy |
| Uptake, Retention, and Adherence to Pre-exposure Prophylaxis (PrEP) in TRIUMPH: A Peer-Led PrEP Demonstration Project for Transgender Communities in Oakland and Sacramento, California | Sevelius et al, 2021  Journal of Acquired Immune Deficiency Syndromes | mixed population |
| Use of Pre-exposure Prophylaxis (PrEP) in Young Men Who Have  Sex with Men is Associated with Race, Sexual Risk Behavior and  Peer Network Size | Kuhns et al., 2017  AIDS and Behavior | not PrEP use |
| Embedding a Linkage to Preexposure Prophylaxis Care Intervention in Social Network Strategy and Partner Notification Services: Results From a Pilot Randomized Controlled Trial | da Silva et al.,2021  Journal of Acquired Immune Deficiency Syndromes | mixed population |
| Population-level effectiveness of rapid, targeted,  high-coverage roll-out of HIV pre-exposure prophylaxis in  men who have sex with men: the EPIC-NSW prospective  cohort study | Grulich et al., 2018  Lancet HIV | mixed population |
| FACTORS ASSOCIATED WITH LOSS TO RETENTION AMONG FEE-BASED AND FREE PrEP CLIENTS IN THAILAND | Chinbunchorn et al., 2020  Abstract | mixed population |
| Acceptability of an open-label wait-listed trial design: Experiences from the PROUD PrEP study | Gafos et al., 2017  Plos One | not outcome  not PrEP use |

| **Reference** | **D1** | **D2** | **D3** | **D4** | **D5** | **D6** | **D7** | **D8** | **Yes´s score** | **Quality** |
| --- | --- | --- | --- | --- | --- | --- | --- | --- | --- | --- |
| Grinztejn et al., 2018 | Yes | UN | Yes | Yes | NA | NA | No | NA | 3 | moderate |
| Myers et al., 2019 | UN | Yes | Yes | Yes | NA | NA | No | NA | 3 | moderate |
| Rolle et al, 2017 | UN | Yes | Yes | Yes | NA | NA | No | NA | 3 | moderate |
| Dourado et al., 2021 | Yes | Yes | UN | UN | NA | NA | Yes | NA | 3 | moderate |
| Fennel et al., 2019 | Yes | Yes | UN | UN | NA | NA | No | NA | 2 | low |
| Fields et al., 2019 | Yes | Yes | UN | UN | NA | NA | No | NA | 2 | low |
| Serota et al., 2019 | Yes | Yes | Yes | Yes | NA | NA | No | NA | 4 | high |
| Tun et al., 2021 | Yes | UN | UN | UN | NA | NA | Yes | NA | 2 | low |
| Wahome et al., 2020 (2) | Yes | Yes | UN | Yes | NA | NA | Yes | NA | 4 | high |
| Wheeler et al., 2019 | Yes | Yes | Yes | Yes | NA | NA | Yes | NA | 5 | high |
| Phanuphak et al., 2018 | No | UN | Yes | Yes | NA | NA | Yes | NA | 3 | moderate |
| Jallil et al., 2022 | Yes | Yes | UN | Yes | NA | NA | Yes | NA | 4 | high |
| Magno et al., 2022 | Yes | Yes | UN | UN | NA | NA | Yes | NA | 3 | moderate |
| Wu et al., 2022 | Yes | Yes | Yes | UN | NA | NA | UN | NA | 3 | moderate |
| Lin et al., 2022 | Yes | Yes | Yes | Yes | NA | NA | Yes | NA | 5 | high |
| Kimani et al., 2021 | UN | UN | Yes | No | NA | NA | Yes | NA | 2 | low |
| Konda et al., 2022 | Yes | Yes | Yes | Yes | NA | NA | UN | NA | 4 | high |

*Note: UN - unclear; NA-not applicable*

**Appendix 4 -** Risk of bias recruitment of observational studies included studies at systematic review, 2022.

| Reference | 1 | 2 | 3 | 4 | 5 | 6 | 7 | 8 | 9 | 10 | 11 | 12 | 13 | Yes´s score | Quality |
| --- | --- | --- | --- | --- | --- | --- | --- | --- | --- | --- | --- | --- | --- | --- | --- |
| Hosek et al., 2017 | No | No | UN | NA | NA | NA | Yes | Yes | No | Yes | UN | NA | Yes | 4 | moderate |
| Hosek et al., 2013 | Yes | Yes | UN | NA | NA | NA | Yes | Yes | UN | Yes | UN | NA | Yes | 6 | moderate |
| Mayer et al., 2016 | Yes | Yes | Yes | NA | NA | NA | Yes | Yes | UN | Yes | UN | NA | Yes | 7 | high |
| Liu et al., 2017 | UN | UN | No | NA | NA | NA | No | Yes | UN | Yes | UN | NA | Yes | 3 | low |
| Molina et al., 2015 | Yes | Yes | Yes | NA | NA | NA | No | Yes | Yes | Yes | UN | NA | Yes | 7 | high |
| Songtaweesin et al., 2020 | Yes | UN | Yes | NA | NA | NA | No | Yes | UN | Yes | Yes | NA | Yes | 6 | moderate |
| Mayer et al., 2020 | Yes | Yes | No | NA | NA | NA | Yes | Yes | UN | No | UN | NA | Yes | 5 | moderate |
| Grohskopf et al., 2013 | Yes | Yes | Yes | NA | NA | NA | Yes | Yes | Yes | UN | UN | NA | Yes | 7 | high |
| Wirtz et al., 2020 | No | No | UN | NA | NA | NA | No | No | UN | No | Yes | NA | Yes | 2 | low |
| McCormack et al., 2016 | Yes | No | Yes | NA | NA | NA | No | Yes | Yes | No | UN | NA | Yes | 5 | moderate |
| Schneider et al., 2021 | Yes | No | Yes | NA | NA | NA | Yes | Yes | UN | Yes | Yes | NA | Yes | 7 | high |
| Young et al., 2017 | Yes | No | Yes | NA | NA | NA | UN | Yes | UN | Yes | Yes | NA | No | 5 | moderate |

*Note: UN - unclear; NA-not applicable*

**Appendix 5 -** Risk of bias recruitment of interventional studies (trials) included studies at systematic review, 2022.

| **Reference** | 1 | 2 | 3 | 4 | 5 | 6 | 7 | 8 | 9 | 10 | 11 | Yes´s score | Quality |
| --- | --- | --- | --- | --- | --- | --- | --- | --- | --- | --- | --- | --- | --- |
| Grinztejn et al., 2018 | NA | Yes | No | Yes | No | Yes | Yes | Yes | Yes | Yes | NA | 7 | high |
| Lalley-Chareczko et al., 2018 | NA | UN | No | No | No | Yes | Yes | Yes | Yes | No | NA | 4 | moderate |
| Myers et al., 2019 | NA | Yes | No | Yes | No | Yes | UN | Yes | Yes | Yes | NA | 6 | moderate |
| Rolle et al, 2017 | NA | Yes | No | No | No | Yes | UN | Yes | No | Yes | NA | 4 | moderate |
| Laurent et al., 2021 | NA | Yes | Yes | Yes | No | Yes | UN | UN | No | No | NA | 4 | moderate |
| Tun et al., 2021 | NA | Yes | Yes | UN | UN | UN | UN | UN | No | No | NA | 2 | low |
| Wahome et al., 2020 (2) | NA | UN | No | Yes | No | Yes | Yes | Yes | Yes | Yes | NA | 6 | moderate |
| Wheeler et al., 2019 | NA | Yes | UN | No | UN | Yes | Yes | Yes | Yes | No | NA | 5 | moderate |
| Phanuphak et al., 2018 | NA | Yes | UN | UN | UN | Yes | Yes | Yes | Yes | Yes | NA | 6 | moderate |
| Hovaguimian et al., 2022 | NA | Yes | Yes | UN | UN | No | Yes | Yes | Yes | Yes | NA | 6 | moderate |
| Jallil et al., 2022 | NA | Yes | UN | UN | No | Yes | Yes | Yes | Yes | Yes | NA | 6 | moderate |
| Traikiatphum et al., 2022 | NA | Yes | UN | No | UN | No | Yes | No | No | Yes | NA | 3 | low |
| Thongsak et al., 2022 | NA | Yes | Yes | No | UN | UN | Yes | No | Yes | No | NA | 4 | moderate |
| Wu et al., 2022 | NA | No | No | No | UN | No | UN | No | Yes | UN | NA | 1 | low |
| Kimani et al., 2021 | NA | No | No | Yes | No | No | No | Yes | UN | UN | NA | 2 | low |
| Konda et al., 2022 | NA | Yes | UN | No | UN | Yes | Yes | Yes | Yes | Yes | NA | 6 | moderate |

*Note: UN - unclear; NA-not applicable*

**Appendix 6 -** Risk of bias retention of observational studies included studies at systematic review, 2022.

| **Reference** | **1** | **2** | **3** | **4** | **5** | **6** | **7** | **8** | **9** | **10** | **11** | **12** | **13** | **Yes´s score** | **Quality** |
| --- | --- | --- | --- | --- | --- | --- | --- | --- | --- | --- | --- | --- | --- | --- | --- |
| Hosek et al., 2017 | No | No | UN | NA | NA | NA | Yes | Yes | No | Yes | UN | NA | Yes | 4 | moderate |
| Hosek et al., 2013 | Yes | Yes | UN | NA | NA | NA | Yes | Yes | UN | Yes | UN | NA | Yes | 5 | moderate |
| Mayer et al., 2016 | Yes | Yes | Yes | NA | NA | NA | Yes | Yes | UN | Yes | UN | NA | Yes | 6 | moderate |
| Liu et al., 2017 | No | No | No | NA | NA | NA | UN | Yes | UN | Yes | UN | NA | Yes | 3 | low |
| Molina et al., 2015 | Yes | Yes | Yes | NA | NA | NA | No | Yes | Yes | Yes | UN | NA | Yes | 7 | high |
| Songtaweesin et al., 2020 | Yes | UN | Yes | NA | NA | NA | No | Yes | UN | Yes | Yes | NA | Yes | 6 | moderate |
| Mayer et al., 2020 | Yes | Yes | No | NA | NA | NA | Yes | Yes | UN | No | UN | NA | Yes | 5 | moderate |
| Grohskopf et al., 2013 | Yes | Yes | Yes | NA | NA | NA | Yes | Yes | Yes | UN | UN | NA | Yes | 7 | high |
| Wirtz et al., 2020 | No | No | UN | NA | NA | NA | No | No | UN | No | Yes | NA | Yes | 2 | low |
| McCormack et al., 2016 | Yes | No | Yes | NA | NA | NA | No | Yes | Yes | No | UN | NA | Yes | 5 | moderate |
| Schneider et al., 2021 | Yes | No | Yes | NA | NA | NA | Yes | Yes | UN | Yes | Yes | NA | Yes | 7 | high |

*Note: UN - unclear; NA-not applicable*

**Appendix 7 -** Risk of bias retention of interventional studies (trials) included studies at systematic review, 2022.

**Appendix 8.** Meta-regression according to selected covariates.

| **Subgroup** | **Number of studies** | | **Estimate** | | **95% CI** | | **p value** | |
| --- | --- | --- | --- | --- | --- | --- | --- | --- |
| **Demand creation** | | | | | | | | |
| *Study design* |  | |  | |  | |  | |
| RCT | 3 | | - | | - | | - | |
| Cohort | 5 | | -0.57 | | -1.09 to -0.04 | | 0.039 | |
| Cross-sectional | 3 | | -0.61 | | -1.60 to 0.38 | | 0.163 | |
| *Sample size* |  | |  | |  | |  | |
| ≤ 400 | 5 | | - | | - | | - | |
| > 400 | 6 | | -0.23 | | -0.80 to 0.33 | | 0.310 | |
| *Region* |  | |  | |  | |  | |
| Asia | 4 | | - | | - | | - | |
| Western | 7 | | 0.17 | | -0.45 to 0.80 | | 0.482 | |
| *Setting* |  | |  | |  | |  | |
| HIV prevention and care | 6 | | - | | - | | - | |
| Population | 5 | | -0.01 | | -0.84 to 0.81 | | 0.964 | |
| *Risk of bias* |  | |  | |  | |  | |
| Low | 4 | | - | | - | | - | |
| Moderate | 7 | | 0.19 | | -0.35 to 0.73 | | 0.384 | |
| **I^2^** | **98.9%** | | | | | | | |
| **p** | **<0.001** | | | | | | | |
| **R^2^** | **72.8** | | | | | | | |
| **Subgroup** | | **Number of studies** | | **Estimate** | | **95% CI** | | **p value** |
| **Retention** | | | | | | | | |
| *Study design* | |  | |  | |  | |  |
| RCT | | 3 | | - | | - | | - |
| Cohort | | 7 | | -0.04 | | -2.37 to 2.28 | | 0.847 |
| *Sample size* | |  | |  | |  | |  |
| ≤ 400 | | 5 | | - | | - | | - |
| > 400 | | 5 | | 0.08 | | -3.12 to 3.28 | | 0.804 |
| *Region* | |  | |  | |  | |  |
| Asia | | 4 | | - | | - | | - |
| Western | | 6 | | 0.14 | | -1.87 to 2.16 | | 0.538 |
| *Setting* | |  | |  | |  | |  |
| HIV prevention and care | | 7 | | - | | - | | - |
| Population | | 3 | | -0.37 | | -3.30 to 2.57 | | 0.358 |
| *Monitoring* | |  | |  | |  | |  |
| Monthly | | 6 | | - | | - | | - |
| 2-3 months | | 3 | | 0.03 | | -2.30 to 2.36 | | 0.897 |
| 6 months | | 1 | | 0.54 | | -3.47 to 4.55 | | 0.337 |
| *Risk of bias* | |  | |  | |  | |  |
| Low | | 4 | | - | | - | | - |
| Moderate | | 6 | | 0.28 | | -3.84 to 4.40 | | 0.546 |
| High | | 1 | | 0.17 | | -6.28 to 6.61 | | 0.796 |
| **I^2^** | | **0.2%** | | | | | | |
| **p** | | **<0.001** | | | | | | |
| **R^2^** | | **100.0%** | | | | | | |
